# Novel Insights into the Molecular Mechanisms Underlying Robustness and Stability in Probiotic Bifidobacteria

**DOI:** 10.1128/aem.00082-23

**Published:** 2023-02-21

**Authors:** Marie Schöpping, Anisha Goel, Kristian Jensen, Ricardo Almeida Faria, Carl Johan Franzén, Ahmad A. Zeidan

**Affiliations:** a Systems Biology, R&D Discovery, Chr. Hansen A/S, Hørsholm, Denmark; b Division of Industrial Biotechnology, Department of Biology and Biological Engineering, Chalmers University of Technology, Gothenburg, Sweden; c Process Upscaling, Chr. Hansen A/S, Hørsholm, Denmark; d Biochemical Assays, Chr. Hansen A/S, Hørsholm, Denmark; INRS Armand-Frappier Sante Biotechnologie Research Centre

**Keywords:** probiotic bacteria, Bifidobacterium animalis subsp. *lactis*, *Bifidobacteria longum* subsp. *longum*, robustness, stability, transcriptomic, metabolite production, amino acids utilization and synthesis, cell membrane fatty acid profile, cell surface hydrophobicity

## Abstract

Some probiotic bifidobacteria are highly robust and shelf-stable, whereas others are difficult to produce, due to their sensitivity to stressors. This limits their potential use as probiotics. Here, we investigate the molecular mechanisms underlying the variability in stress physiologies of Bifidobacterium animalis subsp. *lactis* BB-12 and Bifidobacterium longum subsp. *longum* BB-46, by applying a combination of classical physiological characterization and transcriptome profiling. The growth behavior, metabolite production, and global gene expression profiles differed considerably between the strains. BB-12 consistently showed higher expression levels of multiple stress-associated genes, compared to BB-46. This difference, besides higher cell surface hydrophobicity and a lower ratio of unsaturated to saturated fatty acids in the cell membrane of BB-12, should contribute to its higher robustness and stability. In BB-46, the expression of genes related to DNA repair and fatty acid biosynthesis was higher in the stationary than in the exponential phase, which was associated with enhanced stability of BB-46 cells harvested in the stationary phase. The results presented herein highlight important genomic and physiological features contributing to the stability and robustness of the studied *Bifidobacterium* strains.

**IMPORTANCE** Probiotics are industrially and clinically important microorganisms. To exert their health-promoting effects, probiotic microorganisms must be administered at high counts, while maintaining their viability at the time of consumption. In addition, intestinal survival and bioactivity are important criteria for probiotics. Although bifidobacteria are among the most well-documented probiotics, the industrial-scale production and commercialization of some *Bifidobacterium* strains is challenged by their high sensitivity to environmental stressors encountered during manufacturing and storage. Through a comprehensive comparison of the metabolic and physiological characteristics of 2 *Bifidobacterium* strains, we identify key biological markers that can serve as indicators for robustness and stability in bifidobacteria.

## INTRODUCTION

Bifidobacteria are important inhabitants of the gastrointestinal tract of humans and animals. Owing to their well-documented health-promoting effects, multiple *Bifidobacterium* strains are used as probiotics in food products and dietary supplements. According to the widely accepted definition, probiotics are “live microorganisms which when administered in adequate amounts confer a health benefit on the host” (1). This definition implies that commercial probiotics, such as bifidobacteria, must maintain their viability throughout the production process and subsequent storage until their administration. During industrial-scale production, probiotic bacteria are exposed to various environmental stressors that may challenge their survival and functionality (2). Therefore, robustness (i.e., “the ability of a strain to sustain its functionality despite being exposed to perturbations” [3]) and stability (i.e., “the ability of a strain to remain viable under given environmental conditions during storage” [3]) are key attributes that need to be considered when selecting probiotic *Bifidobacterium* strains and preparing them for industrial-scale production and commercialization (3). Both attributes are complex phenotypic properties that can be influenced by multiple cellular traits.

*Bifidobacterium* strains differ greatly in robustness and stability (4–8). The survival of strains upon exposure to stress has been linked to various physiological and cell envelope characteristics, like the expression of stress-associated genes and cell membrane fatty acid profiles (3). However, little is known about the cellular traits that render some *Bifidobacterium* strains inherently more robust and stable than other strains.

In recent years, transcriptomics and proteomics have been frequently applied to investigate the response of *Bifidobacterium* strains to various stressors. Several molecular players of the stress response in bifidobacteria have been identified (9–16). For example, a study on the oxidative stress response of B. longum BBMN68 highlighted the importance of the DNA-binding protein from starved cells (Dps, referred to as DNA-binding ferritin-like protein) in DNA protection and survival under aerobic condition (15). Other studies applied differential proteome analyses to compare stress-resistant derivatives, after adaptive laboratory evolution, to their parental strains under non-stressed conditions (11, 12, 17). However, mechanisms identified in adaptive laboratory evolution experiments might not correspond to naturally selected traits that confer robustness and stability.

An alternative approach to gaining mechanistic understanding of stress tolerance is a comprehensive comparison of metabolic and physiological characteristics of strains of different species that intrinsically differ in their robustness and stability. Applying transcriptomics in combination with classical physiological characterization of the cells could provide a valuable means for system-wide understanding of the molecular determinants of robustness and stability in bifidobacteria. Integrating omics data with additional physiological characterization data, such as metabolite profiles and cell surface characteristics, of the studied strains has proved extremely useful in supporting or validating omics-driven hypotheses, and the general interpretation of the data (10–12). Moreover, the approach may result in the identification of putative biomarkers that could serve as predictors for high stability and robustness.

In this study, we compare the metabolic and physiological characteristics of the 2 industrially and clinically relevant strains Bifidobacterium animalis subsp. *lactis* BB-12 (hereafter referred to as BB-12) and B. longum subsp. *longum* BB-46 (hereafter referred to as BB-46), to investigate the molecular mechanisms underlying the variation in their robustness and stability. BB-12 is an exceptionally stress tolerant and stable strain (7, 8), whereas Bifidobacterium longum strains are generally known to be more sensitive to stressors (7, 8). The 2 strains were cultivated in lab-scale pH-controlled fermentations in a recently developed chemically defined medium (CDM) (18), and compared in terms of their growth, substrate consumption, metabolite production, cell envelope characteristics, and short-term survivability. In addition, global and differential gene expression analyses were applied to obtain a detailed picture of their phenotypes, and identify potential transcriptional signatures associated with robustness and stability.

## RESULTS

### Growth and metabolite profiles.

**FIG 1:**
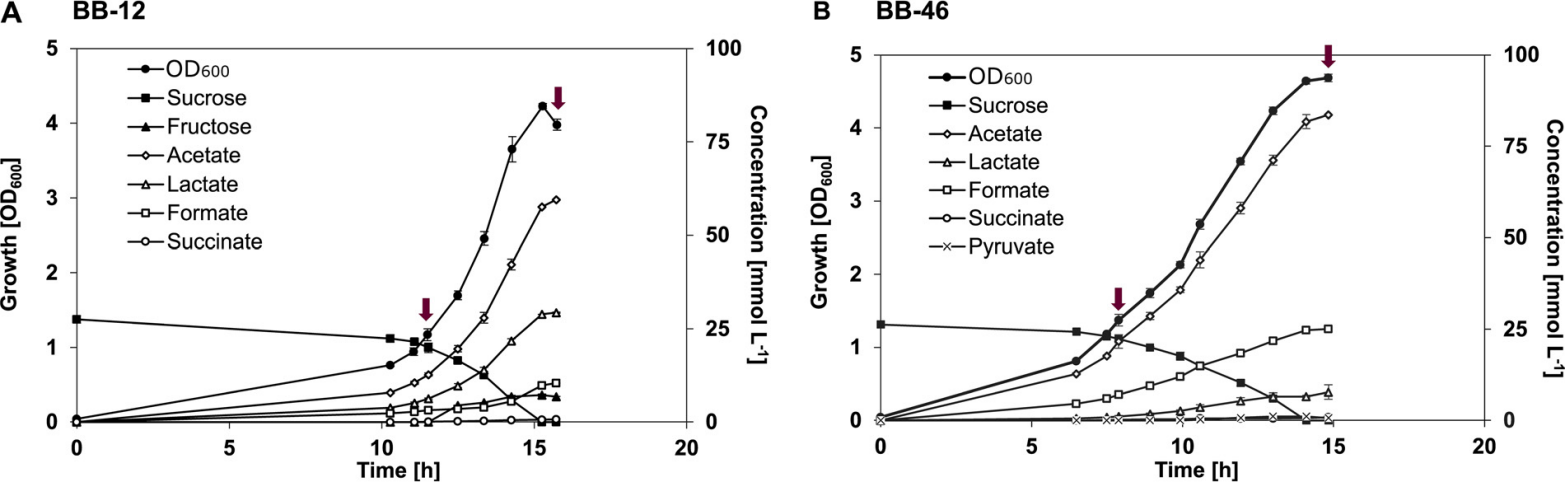
Growth and metabolite profiles of *B. animalis* BB-12 (A) and B. longum BB-46 (B). Cultivations were conducted under anaerobic conditions (80% N_2_ and 20% CO_2_) at 37°C and pH 6.5. Each data point represents the mean of 3 biological replicates ± standard deviation. The arrows indicate the sample time point in the exponential and stationary phase for stability assessment, fatty acid, and transcriptomic analyses.

BB-12 and BB-46 showed different growth dynamics and metabolite profiles when cultivated in the CDM (Fig. 1). BB-12 showed a higher maximum specific growth rate than BB-46, but a slightly lower final biomass yield (Table 1). Moreover, BB-12 grew exponentially until reaching an OD_600_ of ca. 3.6, whereas the growth rate of BB-46 started to decrease already around an OD_600_ of 1.3. The main fermentation products of BB-12 were acetate and lactate, whereas minor amounts of formate and succinate were detected. In contrast, BB-46 secreted acetate as its main fermentation product, while producing less lactate and more formate than BB-12, reaching a final acetate:lactate ratio almost 6 times higher than BB-12 (Table 1).

**TABLE 1:** Growth rate and final metabolic yields of *B. animalis* BB-12 and B. longum BB-46 grown on CDM^*a*^ (18)

Strain	μ_max_ [h^−1^]	q_Suc_^*b*^ [mmol g_CDW_^−1^ h^−1^]	Yield on sucrose [C-mol C-mol^−1^]							Acetate:lactate [mol:mol]	C-recovery^*c*^ [%]	DoR recovery^*d*^ [−]
			Biomass	Acetate	Lactate	Formate	Succinate	Pyruvate	Fructose			
BB-12	0.42 ± 0.02	5.2 ± 0.3	0.28 ± 0.01	0.35 ± 0.01	0.27 ± 0.00	0.03 ± 0.00	0.01 ± 0.00	-	0.12 ± 0.00	1.95 ± 0.03	1.06 ± 0.02	1.03 ± 0.02
BB-46	0.36 ± 0.02	2.4 ± 0.1	0.33 ± 0.00	0.52 ± 0.01	0.07 ± 0.01	0.08 ± 0.00	0.01 ± 0.00	0.01 ± 0.00	-	11.04 ± 1.84	1.02 ± 0.3	0.98 ± 0.03

a For the calculation of the biomass yield, the molecular mass of biomass was considered to be 24.7 g C-mol^−1^ (18). The final biomass was included in the calculation of the C- and DoR recoveries.

b q_Suc_, specific uptake rate of sucrose.

c C-recovery, carbon recovery.

d DoR-recovery, Degree of Reduction recovery.

**FIG 2:**
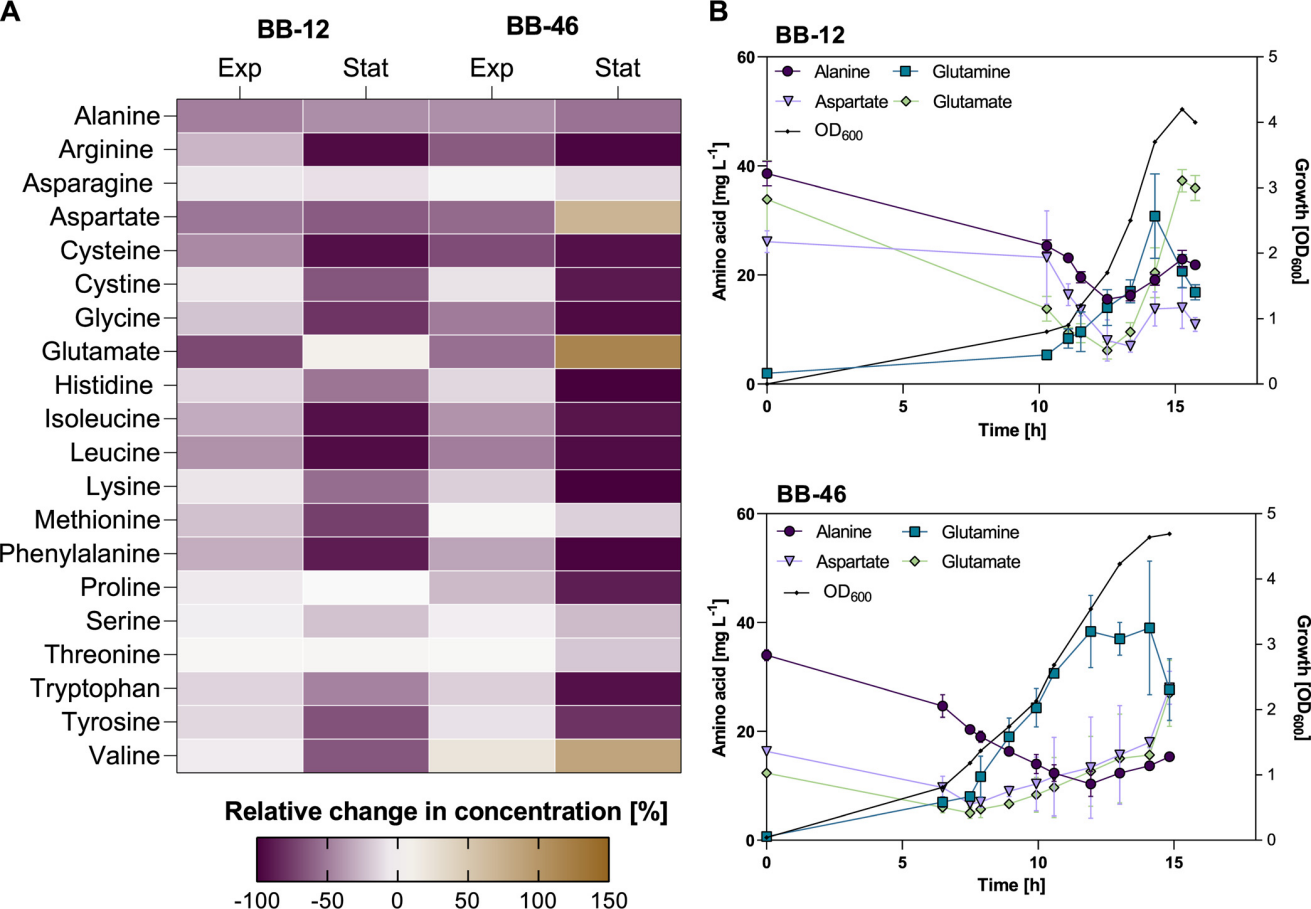
Comparison of amino acid consumption and release by *B. animalis* BB-12 and B. longum BB-46 during growth in chemically defined medium. The chemically defined medium contained all proteinogenic amino acids, except for cysteine in a concentration of 0.04 g L^−1^. Cysteine that served as amino acid, reducing agent and sulfur source was added in a concentration of 0.5 g L^−1^. (A) Relative changes of amino acid concentrations at the exponential and stationary phase. Exp, relative change in concentration between inoculation and exponential phase (around OD_600_ = 1.3). Stat, relative change in concentration between inoculation and stationary phase. Each field represents the relative difference between the mean of 3 biological replicates. Cystine represents the oxidized form of cysteine, presumably formed when cysteine reduces remaining oxygen in the medium before inoculation (concentration at time of inoculation: 0.06–0.07 g L^−1^). (B) Concentrations of L-aspartate, l-glutamate, l-alanine, and l-glutamine during fermentations of BB-12 and BB-46. Each data point represents the mean of 3 biological replicates ± standard deviation.

Most of the 20 free proteinogenic amino acids supplied in the CDM (18) were continuously consumed by both strains throughout growth, with most of them being nearly depleted when the strains reached their stationary phase (Fig. 2A). However, only minor decreases were detected in the concentrations of l-asparagine, l-serine, and l-threonine for both strains, as well as of l-methionine for BB-46 and L-proline for BB-12 (Fig. 2A). BB-12 and BB-46 secreted l-alanine, l-glutamate, and L-aspartate (Fig. 2B). BB-12 consumed l-valine, whereas BB-46 secreted l-valine until entering the stationary phase (Fig. 2A). Despite being added to the medium, l-glutamine was not detected in the initial medium, possibly due to degradation during heat sterilization of the amino acid solution. The concentration of l-glutamine gradually increased in the fermentation medium of both strains until the end of the log phase, when it decreased slightly (Fig. 2B).

L-Cysteine was used as a reducing agent, and, thus, added in a significantly higher concentration to the CDM than the other amino acids (0.5 g L^−1^ versus 0.04 g L^−1^ for other amino acids). Besides cysteine, its dimer cystine was detected in the culture medium. Both metabolites were almost depleted at the end of the fermentation (Fig. 2A).

### Cell viability following short-term storage.

**FIG 3:**
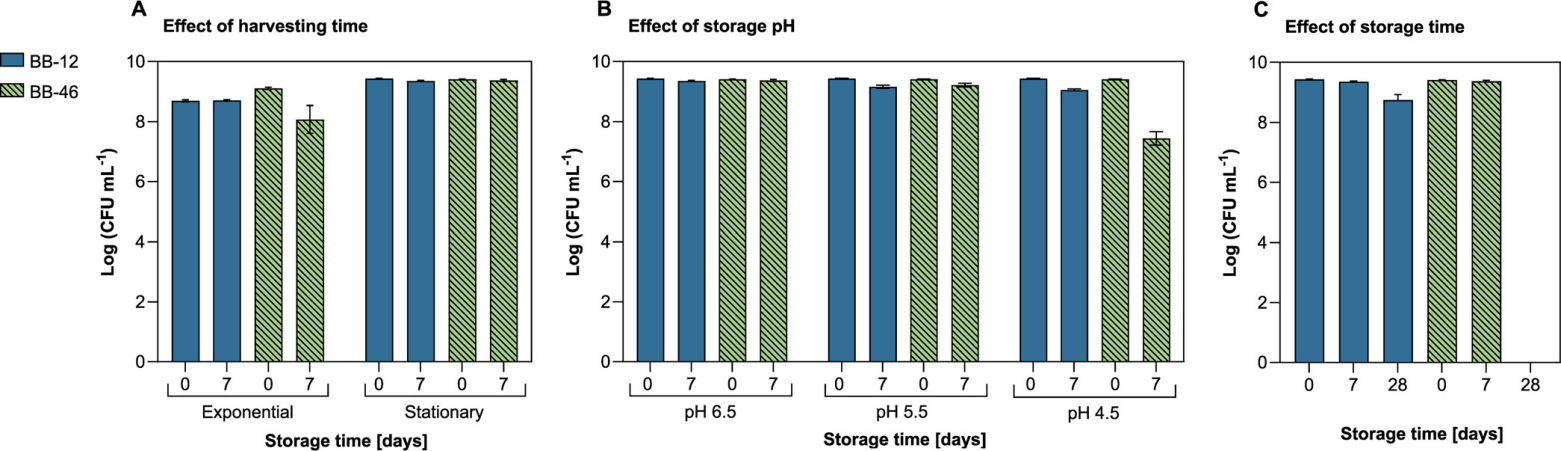
Viability assessment of *B. animalis* BB-12 and B. longum BB-46 during short-term storage. Cells were stored under aerobic conditions for 7 to 28 days at 8 to 10°C at different pH (pH 6.5, pH 5.5, and pH 4.5), and their viability was assessed by CFU counts before and after storage. Each data point represents the mean of 3 biological replicates ± standard deviation. (A) Effect of harvesting time. Viability of BB-12 and BB-46 harvested in the exponential and stationary phase before and after storage for 7 days at pH 6.5. (B) Effect of storage pH. Viability of BB-12 and BB-46 harvested in the stationary phase before and after storage at pH 6.5, pH 5.5, and pH 4.5 for 7 days. (C) Effect of storage time. Viability of BB-12 and BB-46 harvested in the stationary phase before and after storage at pH 6.5 for 7 days or 28 days.

Previous studies have shown that BB-12 is relatively robust, whereas BB-46 is relatively sensitive toward the presence of environmental stressors. However, since cultivation conditions can affect the stress physiology of the produced strains (19), their robustness and stability were assessed in short-term storage tests when being produced under the applied cultivation conditions. The storage conditions were selected to assess the tolerance of the strains toward various stressors, including low temperature (8 to 10°C), oxidative stress (aerobic storage condition), and elevated temperature (contact with 46 ± 1°C molten agar medium). In addition to these consistent treatments, the effect of the harvesting time point, the storage pH, and the storage time on their viability was assessed (Fig. 3). Thereby, also the effects of starvation and acidic stress were assessed (storage pH of 5.5 and 4.5).

The number of viable BB-12 cells remained almost unchanged when stored for 7 days at pH 6.5, regardless of whether the cells had been harvested in the exponential or stationary phase (Fig. 3A). In contrast, the harvesting time point affected the survivability of BB-46 when stored under the same conditions. A total of 90% of the BB-46 cells that had been harvested in the exponential phase lost viability (1.0 log_10_ loss), whereas no viability loss was detected for cells harvested in the stationary phase (Fig. 3A). The number of viable cells after 7 days decreased for both strains with decreasing storage pH from pH 6.5 to pH 4.5 (Fig. 3B). Both strains demonstrated similar survival at pH 6.5 and pH 5.5. However, at pH 4.5, the viability loss of BB-46 was higher than that of BB-12 (Fig. 3B). Moreover, no viable BB-46 cells were detected after storage for 28 days at pH 6.5, corresponding to a viability loss of 9.2 log_10_ CFU mL^−1^, whereas BB-12 showed a viability loss of 0.7 log_10_ CFU mL^−1^, or 80% (Fig. 3C). Cells of BB-12 showed strong sedimentation during storage, whereas cells of BB-46 remained evenly distributed throughout the suspension.

For BB-12, the drop in active cell numbers, as determined by flow cytometric measurement of the membrane potential, agreed with the viable cell count based on CFU (Table S1). In contrast, the active cell count of BB-46 exceeded the CFU count determined after storage under some conditions, particularly after storage at pH 4.5 for 7 days and at pH 6.5 for 28 days, which is reflected by a higher loss of viability than of activity under these conditions (Table S1).

### Global and differential gene expression analysis.

The transcriptome analysis was applied to investigate the molecular mechanisms underlying the higher stability and robustness of BB-12 and the improved stress resistance of BB-46 in the stationary phase. The data was analyzed in the context of metabolic pathways. All significantly, differentially, expressed genes were searched for that have been previously described to be involved in the stress response of bifidobacteria (3, 20), or that are commonly known to be involved in the defense mechanism of bacteria.

**FIG 4:**
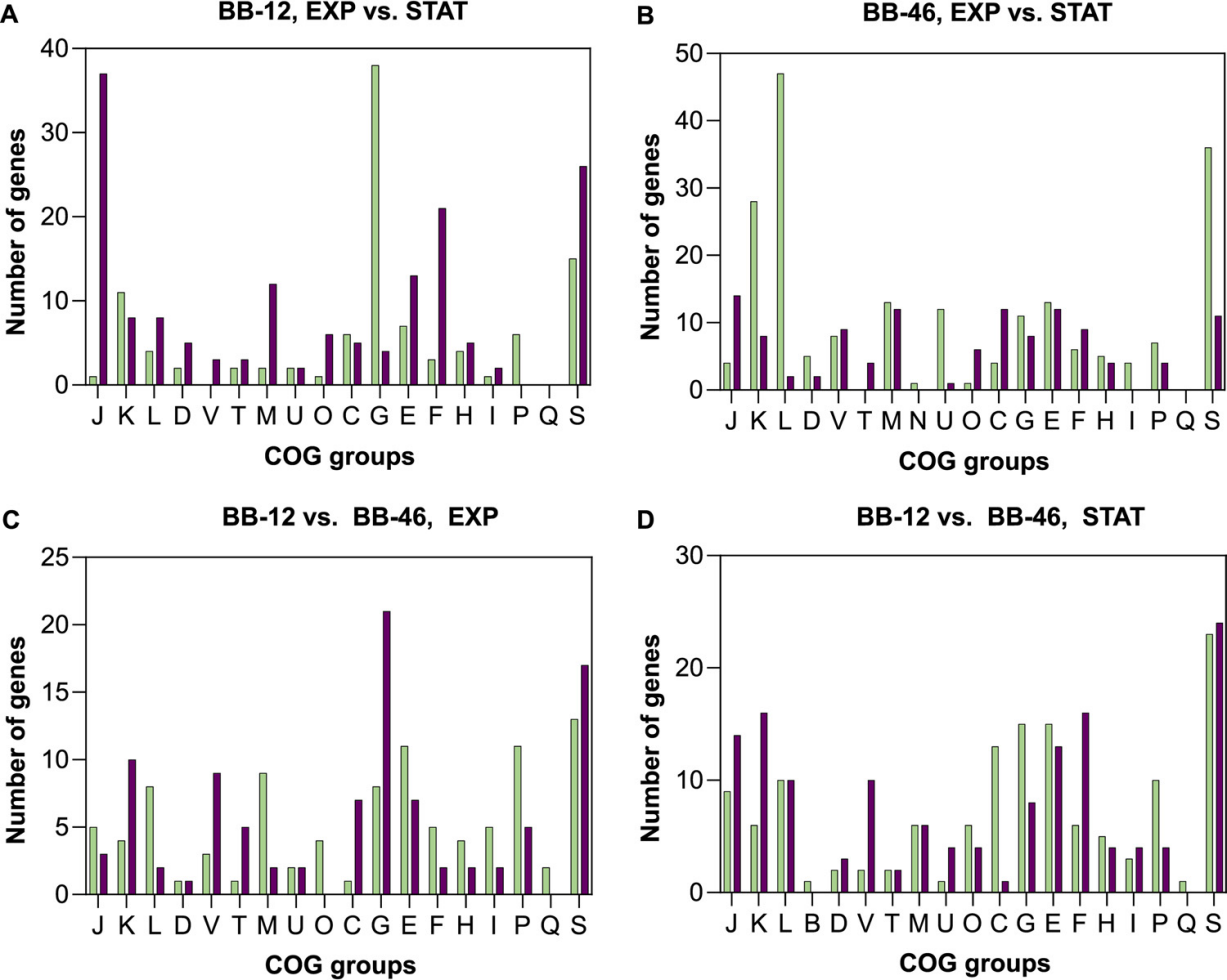
COG classification of significantly differentially expressed genes. (A and B) Differentially expressed genes between the exponential and stationary phase in BB-12 and BB-46, respectively. (A) 43 genes, and (B) 151 genes not assigned to COG groups. Green, upregulated; red, downregulated. (C and D) Differentially expressed genes between BB-12 and BB-46 in the exponential and stationary phase, respectively. (C) 13 genes, and (D) 21 genes not assigned to COG groups. Green, higher expressed in BB-12; red, higher expressed in BB-46. COG Categories: Information Storage and Processing: J, translation, ribosomal structure, and biogenesis; K, transcription; L, replication, recombination, and repair; B, chromatin structure and dynamics. Cellular Processes and Signaling: D, Cell cycle control, cell division, and chromosome partitioning; V, defense mechanisms; T, signal transduction mechanisms; M, cell wall/membrane/envelope biogenesis; N, cell motility; U, intracellular trafficking, secretion, and vesicular transport; O, posttranslational modification, protein turnover, and chaperones. Metabolism: C, energy production and conversion; G, carbohydrate transport and metabolism; E, amino acid transport and metabolism; F, nucleotide transport and metabolism; H, coenzyme transport and metabolism; I, lipid transport and metabolism; P, inorganic ion transport and metabolism; Q, secondary metabolites biosynthesis, transport, and catabolism. Poorly Characterized: S, function unknown.

**(i) Growth phase-specific gene expression.** The genomes of BB-12 and BB-46 include 1550 and 1932 genes, respectively. A total of 318 genes (21%) and 474 genes (25%) were differentially expressed [|log_2_(FC)| ≥ 2 and *P* value ≤ 0.01] in BB-12 and BB-46, respectively, between the exponential and stationary phase (Data set S1 and S2). Differentially expressed genes were grouped into COGs (Fig. 4A and B), highlighting e.g., a strong upregulation of genes assigned to COG G (Carbohydrate transport and metabolism) in BB-12 and of COG L (Replication, recombination, and repair) in BB-46.

In BB-46, 28% of all differentially expressed genes could not be assigned any functional annotation, hampering the interpretation of the growth phase specific gene expression in the strain. In BB-12, only 12% of the differentially expressed genes were hypothetical proteins.

A total of 1132 orthologous genes were identified in BB-12 and BB-46. Comparing the pattern of regulation (up-or downregulation) of these orthologs in BB-12 and BB-46, revealed significantly different growth phase-associated changes for 256 orthologues gene (23%) in the strains, including 11 of 39 orthologous genes annotated as transcriptional regulators (Data set S3).

**(ii) Upregulation of carbohydrate transport and utilization in the stationary phase.** Most genes of the bifid shunt were significantly downregulated in BB-12 and BB-46 in the stationary phase (Data set S1 and S2). However, in BB-12, multiple genes linked to the uptake of carbohydrates were strongly upregulated (COG group G) (Fig. 4A). Besides carbohydrate transporter genes, genes encoding enzymes that convert several carbohydrates into intermediates of the bifid shunt were upregulated in BB-12 (Table S2).

An upregulation of genes associated with carbohydrate utilization was also observed in BB-46 in the stationary phase, however, to a smaller extent than in BB-12 (Table S2).

**FIG 5:**
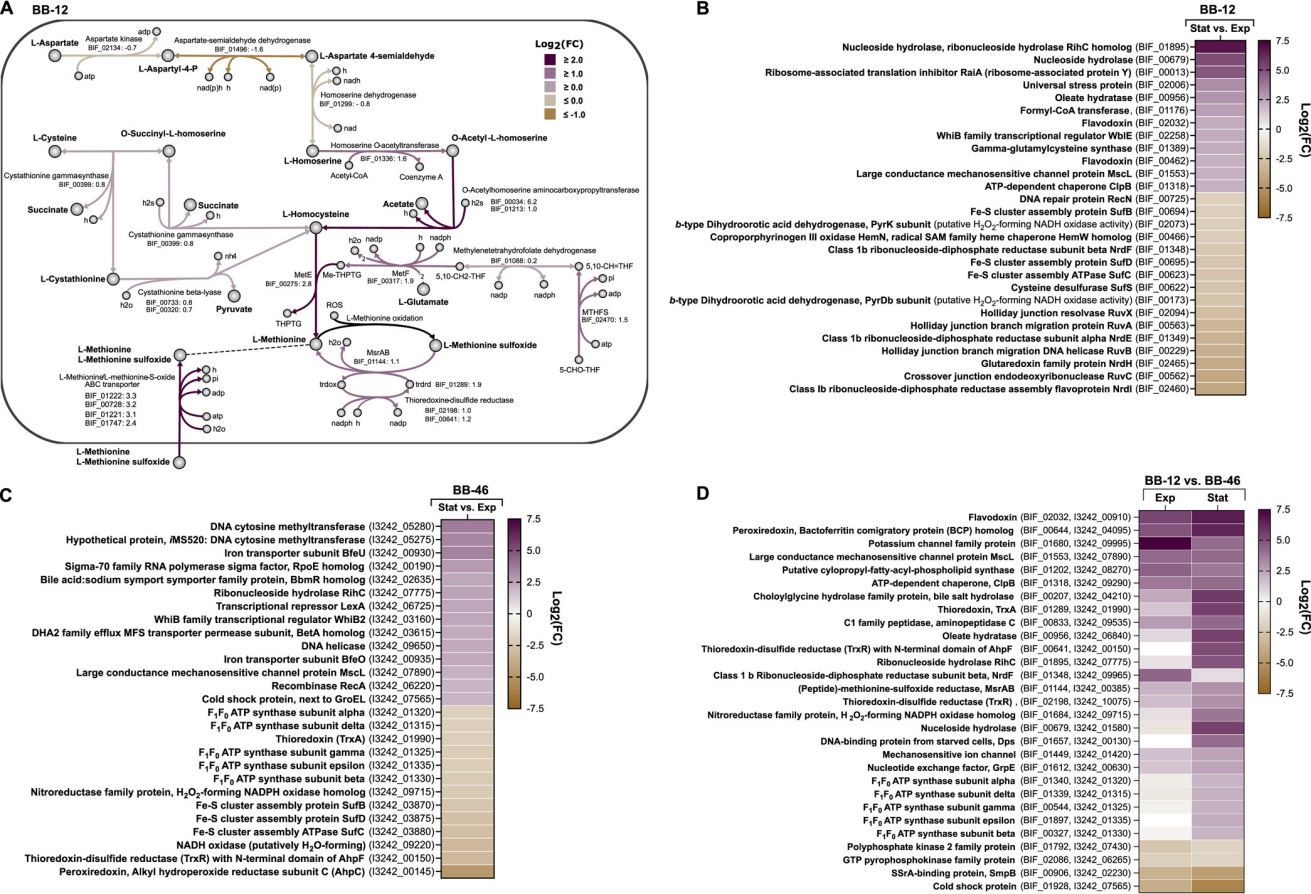
Differential gene expression analysis of *B. animalis* BB-12 and B. longum BB-46 using RNA-seq. (A) Schematic representation of differentially expressed genes associated with methionine biosynthesis in BB-12 between the exponential (Exp) and stationary phase (Stat). The locus tags of all differentially expressed genes are given in bold type next to their potential functionalities. The log_2_(FC) values are given next to the locus tags. All log_2_(FC) values have an adjusted *P* value ≤ 0.01. L-Aspartyl-4-P, L-aspartyl-4-phosphate; MTHFS, 5-formethyltetrahydrofolate cyclo-ligase; MetE, 5-methyltetrahydropteroyltriglutamate–homocysteine methyltransferase; MetF, 5,10-methylenetetrahydrofolate reductase; 5-CHO-THF, 5-formyltetrahydrofolate; THPTG, tetrahydropteroyltri-l-glutamate; h2s, hydrogen sulfide; 5,10-CH=THF, 5,10-Methenyltetrahydrofolate; ME-THPTG, 5-methyltetrahydropteroyltri-l-glutamate; 5,10-CH2-THF, 5,10-methylenetetrahydrofolate; MsrAB, (peptide)-l-methionine-(R/S)-sulfoxide reductase; ROS, reactive oxygen species; trdox, oxidized thioredoxin; trdrd, reduced thioredoxin. (B, C, and D) Differentially expressed genes previously implicated in the stress response of bifidobacteria (3, 20) or commonly known to be involved in defense mechanism of bacteria. (B) In BB-12 between the exponential and stationary phase. |Log_2_(FC)| ≥ 2 and *P* value ≤ 0.01. (C) In BB-46 between the exponential and stationary phase. |Log_2_(FC)| ≥ 2 and *P* value ≤ 0.01. (D) Between BB-12 and BB-46 in the exponential and stationary phase. |Log_2_(FC)| ≥ 2 and *P* value ≤ 0.01. Log_2_(FC) > 0, higher transcription level of gene in BB-12. White fields, no significant difference in the expression level of the gene between the strains.

**(iii) Upregulation of methionine biosynthesis and cycling in BB-12 in the stationary phase.** The transcription level of most genes associated with amino acid biosynthesis decreased in BB-12 in the stationary phase (COG group E) (Fig. 4A), particularly genes involved in l-histidine biosynthesis (BIF_01260, BIF_01259, BIF_02246, BIF_02247, log_2_[FC]: −2.8 to −4.6) (Data set S1). In contrast, multiple genes associated with the biosynthesis of l-methionine from L-homoserine and from l-cysteine were upregulated in the stationary phase in BB-12 (log_2_[FC]: 0.8 to 6.2) (Fig. 5A). Moreover, genes encoding enzymes involved in the supply of 5-methyltetrahydropteroyltri-l-glutamate (Met-THPTG) were upregulated. Met-THPTG acts as a methyl donor of 5-methyltetrathydropteroyltriglutamate-homocysteine S-methyltransferase (MetE) (Fig. 5A). In contrast, genes encoding enzymes that are responsible for the supply of L-homoserine from L-aspartate were slightly downregulated (Fig. 5A).

Methionine biosynthesis from L-homoserine involves the activation of L-homoserine either by L-homoserine O-acetyltransferase or homoserine O-succinyltransferase (21). Prokaryotic genome annotation pipeline (PGAP) annotated the BIF_01336 gene in BB-12 as a homoserine O-succinyltransferase. However, our protein sequence analysis of this gene showed that it carries a glutamate residue at position 111. The same was found to be true for the homolog (I3242_01295) in BB-46. According to previous studies in Bacillus cereus, this suggests that the gene actually encodes a homoserine O-acetyltransferase (21, 22) (Fig. 5A).

Besides genes associated with methionine biosynthesis, the transcription of 4 homologs of genes of the *metNPQ* operon in Bacillus subtilis, encoding an ABC-type transporter that facilitates D-/l-methionine as well as l-methionine sulfoxide uptake, were upregulated significantly in BB-12 (Fig. 5A) (log_2_[FC]: 2.4 to 3.3) (18, 23). In addition, the transcription of a gene annotated by PGAP as peptide-l-methionine-(R)-sulfoxide reductase, MsrB, was slightly upregulated (BIF_01144) (Fig. 5A). Methionine sulfoxide reductases (Msr) catalyze the reduction of methionine sulfoxide to methionine, resulting in a reactive oxygen species (ROS)-scavenging cycle (24). Contrary to the functional annotation by PGAP, we found that the gene in BB-12 and its ortholog in BB-46 (I3242_00385) possess both a MsrA and MsrB domain, which is in line with the KEGG annotation (25). MsrA and MsrB differ in their substrate specificity, acting on free and protein-based l-methionine-S-sulfoxide, and protein-based l-methionine-R-sulfoxide, respectively (24).

In BB-46, the only gene of l-methionine biosynthesis that was significantly upregulated was a gene encoding cystathionine-beta synthase [I3242_02345, log_2_(FC): 2.4], while other genes associated with l-methionine biosynthesis were downregulated.

**(iv) Upregulation of the fatty acid metabolism in BB-46 in the stationary phase.** In BB-46, 4 consecutive genes in the genome that are involved in the fatty acid metabolism were significantly upregulated in the stationary phase (COG group I) (Fig. 4B). Besides a multifunctional type I fatty acid synthase (FAS, I3242_09035, log_2_[FC]: 3.3), the transcription of 2 genes encoding acetyl-CoA carboxylase (I3242_09040, I3242_09045, log_2_[FC]: 3.7, 4.6), and a gene coding for a biotin transporter BioY, were significantly upregulated. The latter was the most upregulated gene in BB-46 between the growth phases (I3242_09050, log_2_[FC]: 7.4). In BB-12, the expression of the corresponding orthologous genes (BIF_00783, BIF_01198 – BIF_01200) was instead slightly downregulated in the stationary phase, while being among the most expressed genes in the exponential phase. Moreover, the transcription of all 4 genes possibly encoding for long-chain fatty acid-CoA ligases in both BB-46 and BB-12, was generally reduced in the stationary phase. However, only one of the 4 genes in BB-12 was significantly downregulated (BIF_00385, log_2_[FC]: −4.0).

**(v) Differential expression of stress-associated genes between growth phases in BB-12 and BB-46.** Multiple stress-associated genes (3, 20) were significantly differentially expressed in the stationary phase than in the exponential growth phase in BB-12 and in BB-46 (Fig. 5B and C).

The transcription level of a gene encoding formyl-CoA transferase (log_2_[FC] = 2.6) increased significantly in BB-12 in the stationary growth phase (Fig. 5B). Formyl-CoA transferase is involved in the catabolism of the organic acid oxalate, present in human gastrointestinal tract (26). Additionally, the gene encoding the second key enzyme of oxalate catabolism, oxalyl-CoA decarboxylase, was upregulated (BIF_00592, log_2_[FC] = 1.6). The 2 genes became the 3rd and 8th most expressed genes in BB-12 in the stationary phase. Furthermore, the transcription of a homolog of the previously described putative oxalate transporter in *B. animalis* subsp. *lactis* BI07 (sequence identity and coverage: 100%) was upregulated (BIF_02925, log_2_[FC]: 1.8) (26).

Two genes encoding nonspecific nucleoside hydrolase were upregulated in BB-12 in the stationary phase (log_2_[FC]: 6.8, 5.1) (Fig. 5B), with one of them becoming the 7th most expressed gene. Similarly, the transcription of its ortholog in BB-46, annotated as ribonucleoside hydrolase RihC, was increased in the stationary phase (log_2_[FC]: 2.5) (Fig. 5C).

In addition, a gene annotated as ribosome-associated translation inhibitor RaiA, was significantly upregulated in BB-12 in the stationary phase (log_2_[FC]: 4.8) (Fig. 5B), becoming the 5th most expressed gene. In contrast, the RaiA ortholog in BB-46 was slightly downregulated in the stationary phase (I3242_06205, log_2_[FC]: −1.2). RaiA is suspected to promote ribosome stabilization under environmental stress (27), and reduce the protein biosynthesis rate due to interference with the ribosomal A site (28).

Stress-related genes whose expression significantly decreased in BB-12 in the stationary growth phase included several genes involved in DNA repair (COG group L) (Fig. 4A and Fig. 5B), such as genes associated with recombinational repair (RuvCAB and RecN) [log_2_(FC): −2.0 to −4.0] (29). In addition, genes of the ribonucleotide reductase gene cluster Ib NrdHIEF were significantly downregulated [log_2_(FC): −2.4 to −4.1]. Ribonucleotide reductases are responsible for the supply of deoxyribonucleotides as precursor of DNA synthesis and repair. Also, genes encoding the PyrK and PyrDb subunits of a *b*-type dihydroorotic acid dehydrogenase (DHODb), known as an enzyme in pyrimidine biosynthesis, were significantly downregulated [log_2_(FC): −2.1, −2.6] (Fig. 5B). The homolog of DHODb in the O_2_-sensitive B. bifidum JCM 1255 has been suggested to act as a hydrogen peroxide (H_2_O_2_) forming NADH oxidase under aerobic conditions (20, 30).

Contrary to the trend in BB-12, multiple genes linked to DNA repair were significantly upregulated in BB-46 (COG Group L) (Fig. 4B and Fig. 5C), including genes predicted to encode DNA-cytosine methyltransferases [log_2_(FC): 3.7, 3.4], the transcriptional repressor LexA [log_2_(FC): 2.4], the DNA helicase [log_2_(FC): 2.3], and the recombinase RecA [log_2_(FC): 2.0] (Fig. 5C). In addition, the transcription of a gene encoding a cold shock protein was significantly upregulated [log_2_(FC): 2.0] (Fig. 5C).

Furthermore, 2 genes potentially involved in bile transport were upregulated in the stationary phase in BB-46. These were: I3242_03615, a homolog of the bile efflux system BetA from B. breve UCC2003 [log_2_(FC): 2.3] (31), and I3242_02635, a gene annotated as bile acid sodium symporter and which is a homolog of the Bifidobacterium breve macrolide Resistance protein BbmR of B. breve UCC2003 [query coverage: 100%, sequence identity: 94.90%, log_2_(FC): 2.4] (Fig. 5C) (30). Moreover, 5 of the 8 genes encoding the H^+^-translocating F_1_F_0_-ATPases (ATP synthase) were significantly downregulated in BB-46 [log_2_(FC): −2.0 to −2.3] (Fig. 5C), while the expression of their orthologs in BB-12 increased slightly in the stationary phase [log_2_(FC): 0.3 to 0.8] (Data set S1).

Several genes linked to the detoxification of ROS were downregulated in the stationary phase in BB-46, including AhpC and TrxR encoding the alkyl hydroperoxide reductase subunit C-thioredoxin-disulfide reductase (AhpC-TrxR) system [log_2_(FC): −4.8, −3.2], a NADH oxidase (putatively H_2_O-forming) [log_2_(FC): −3.2], and TrxA [log_2_(FC): −2.1] (Fig. 5C) (20). In addition, also the transcription of a gene encoding a homolog of the H_2_O_2_-forming NADPH oxidase NPOX of *B. infantis* JCM 1222 (31) was significantly reduced [log_2_(FC): −2.3] (Fig. 5C).

Moreover, the transcription of BfeU and BfeO, 2 adjacent genes annotated as a FTR1 family protein and iron transporter with high sequence identity to proteins of the iron uptake system BfeUO in B. breve UCC2003 (BfeU: 73% and BfeO: 85%) (32), was significantly upregulated in BB-46 in the stationary phase [log_2_(FC): 3.5, 2.3] (Fig. 5C). On the contrary, genes encoding proteins of the SUF iron-sulfur (Fe-S) cluster assembly machinery were significantly downregulated in both strains, including Fe-S cluster assembly proteins SufB [log_2_(FC): −2.5 in BB-12 and −2.1 in BB-46], SufC [log_2_(FC): −2.9 in BB-12 and −2.6 in BB-46], and SufD [log_2_(FC): −2.9 in BB-12 and −2.5 in BB-46], as well as cysteine desulfurase (SufS) [log_2_(FC): −2.6] in BB-12 (Fig. 5B and Fig. 5C).

**(vi) Miscellaneous significantly differentially expressed genes.** As an expected consequence of ceased growth, the transcription of most genes coding for ribosomal proteins and all genes encoding tRNA ligases was downregulated in the stationary phase (Data set S1 and S2).

Moreover, in BB-46, 7% of the genes that were shown as upregulated in the stationary phase are annotated as transposases (Data set S2). However, due to high homology of transposase genes of the same insertion sequence (IS) families, individual log_2_(FC) values might be over- or underestimated. The genome of BB-12 contains only 10 transposase genes, while BB-46 possesses 44 transposase genes. None of BB-12’s transposase genes were upregulated in the stationary phase.

Besides the downregulation of genes encoding NrdHIEF, and the 2 subunits of DHODb in BB-12 (Fig. 5B), multiple additional genes involved in pyrimidine (BIF_00104, BIF_00454, BIF_01872, BIF_01915, BIF_01973) and purine synthesis (BIF_00341, BIF_0506, BIF_00705, BIF_00886, BIF_00947, BIF_00948) were significantly downregulated in the stationary phase (Data set S1). This is in line with the (significant) downregulation of orthologous genes in BB-46 (Data set S2).

A gene annotated as acetoin reductase was the third most upregulated gene in BB-12 in the stationary phase [BIF_02081, log_2_(FC): 7.6]. Finally, several genes annotated as glycosyltransferases were significantly downregulated in both BB-12 and BB-46 in the stationary phase (Data set S1and S2).

**(vii) Strain-specific transcription of stress-associated genes.** Besides comparing the gene expression profile of each strain between growth phases, the expression levels of 1132 orthologous genes in BB-12 and BB-46 were compared in both growth phases using differential gene expression analysis. In total, 207 orthologs (18%) were differentially transcribed in the 2 strains in the exponential phase (Data set S4) and 300 genes (27%) in the stationary phase (Data set S5), with 100 genes (9%) being differentially expressed in the 2 strains across both growth phases [|log_2_(FC)| ≥ 2, *P*_adj_ < 0.01]. The differentially expressed genes were grouped into COGs (Fig. 4C and 4D), revealing e.g., that several genes assigned to COG group O (posttranslational modification, protein turnover, and chaperones) are considerably higher expressed in BB-12 than in BB-46 across the growth phases.

Numerous genes that showed significantly higher expression in BB-12 compared to BB-46 are associated with the response to stress (Fig. 5D), especially with oxidative and osmotic stress, as well as with protein quality control in bifidobacteria (3, 20). Most of the orthologs showed a more pronounced difference in their expression in the stationary growth phase (Fig. 5D).

Several genes involved in ROS-scavenging showed higher expression level in BB-12 than in BB-46 in the exponential, stationary, or both growth phases. These included genes encoding flavodoxin, a bacterioferritin comigratory protein (BCP) homolog, thioredoxin (TrxA), the TrxR of the AhpC-TrxR system, MsrAB, a second TrxR, and Dps [log_2_(FC): 1.5 to 6.6] (Fig. 5D) (20). Four of these genes were among the hundred most expressed genes in BB-12 in the stationary phase (Dps > BCP > TrxR of AhpC-TrxR > TrxA). Also, the gene encoding the NPOX homolog in BB-12 was significantly more expressed than in BB-46 in the stationary phase [log_2_(FC): 3.9]. In addition, across both growth phases, BB-12 showed a notably higher expression of a gene encoding bile salt hydrolase (BSH) [log_2_(FC): 2.3, 5.7], being the 36^th^ and 11th most expressed gene in BB-12 in the exponential and stationary phase, respectively. Also, a gene annotated as oleate hydratase, which has been found to contribute to resistance to organic solvent stress in bifidobacteria (33), appeared higher expressed in the stationary phase in BB-12 than in BB-46 in the stationary phase [log_2_(FC): 5.5] (Fig. 5D). In BB-12, genes that may play a role in osmotic stress responses were consistently higher expressed, such as genes encoding a potassium transporter [log_2_(FC)_BB-12Exp/BB-46Exp_: 4.9, log_2_(FC)_BB-12Stat/BB-46Stat_: 6.4], and a mechanosensitive ion channel [log_2_(FC)_BB-12Stat/BB-46Stat_: 2.5] (Fig. 5D). A large conductance mechanosensitive channel protein MscL was upregulated in both BB-12 and BB-46 in the stationary phase to the same extent [log_2_(FC): 2.1], however, the general transcription level of MscL was significantly higher in BB-12 than in BB-46 in both growth phases [log_2_(FC): 4.3, 4.3] (Fig. 5D). Also, multiple genes encoding F_1_F_0_-ATPase subunits reached a notably higher expression level in BB-12 than in BB-46 in the stationary phase [log_2_(FC): 2.0 to 2.2] due to their significant downregulation in BB-46 and slight upregulation in BB-12 (Fig. 5C and D). Furthermore, genes encoding the 2 heat shock proteins GrpE and ClpB were consistently expressed at higher levels in BB-12 than in BB-46 [log_2_(FC): 1.5 to 4.2] (Fig. 5D). In BB-12, the heat shock proteins ClpB, DnaK, GroEL, and ClpC were among the hundred most expressed genes in both growth phases, which was only the case for GroEL in BB-46 (Data set S1 and S2).

Only a few stress-associated genes showed higher expression in BB-46 than in BB-12 (Fig. 5D). These included a gene annotated as SSrA-binding protein SmpB [log_2_(FC)_BB-12Exp/BB-46Exp_: −3.5, log_2_(FC)_BB-12Stat/BB-46Stat_ −4.5], known to be responsible for releasing stalled ribosomes under stress conditions (34), and a gene encoding a cold shock protein [log_2_(FC): −3.7, −5.9]. Moreover, the expression level of a gene annotated as polyphosphate kinase 2 family protein was considerably higher in the exponential phase in BB-46 than in BB-12 [log_2_(FC): −2.5]. Polyphosphate kinase 2 catalyzes the synthesis of nucleoside triphosphates from polyphosphates, primarily of GTP (35), which serves as a substrate for DNA and RNA synthesis.

### Cell envelope characteristics.

The cell envelope represents the physical barrier between the cytoplasm and the extracellular environment. Therefore, it plays a crucial role in conferring robustness and stability (36, 37). Previous studies have proposed a correlation between the fatty acid composition of the cytoplasmatic membrane of *Bifidobacterium* strains, and their ability to survive environmental stressors (14, 38–40). Therefore, the fatty acid profiles of BB-12 and BB-46 were analyzed in the exponential and stationary phase.

**FIG 6:**
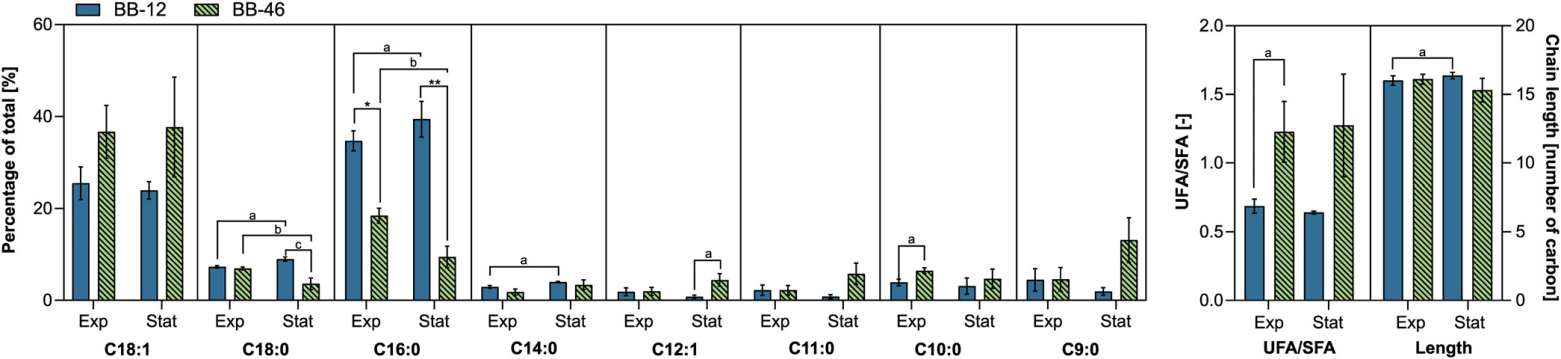
Membrane fatty acid profiles of *B. animalis* BB-12 and B. longum BB-46 showing the main components in exponential (Exp) and stationary phase (Stat). Each data point represents the mean of 3 biological replicates ± standard deviation. UFA/SFA, unsaturated:saturated fatty acid ratio. The significance of the differences between means was assessed in t-tests. Means with the same superscript letters show differences at *P* ≤ 0.05 before multiple testing, while means with the same number of superscript asterisks (*) within the same group show differences at *P*_adj_ ≤ 0.05, after correcting for multiple testing. When superscripts are omitted, no significant difference was observed. The complete fatty acid profile is shown in Table S3.

Significant differences in the relative fatty acid content were detected between growth phases, as well as between strains (*P* ≤ 0.05). However, when correcting for multiple testing, only some differences between the strains remained significant (*P*_adj_ ≤ 0.05) due to a rather high standard deviation of the percentage of some fatty acids in the cell membrane of BB-46 (Fig. 6 and Table S3).

Eight of the 23 identified fatty acids (Fig. 6) reached either proportions above 5% in 1 of the strains, or their proportions varied by more 2% when comparing the fatty acid profile of 1 strain at different growth phases, or the profiles of the 2 strains during a particular growth phase.

The fatty acid profile of the cell membrane of BB-12 was dominated by palmitic acid (C16:0), and oleic acid (C18:1) was the second most abundant in both growth phases (Fig. 6). In BB-46, palmitic acid and oleic acid were also the major fatty acids in the cytoplasmic membrane in the exponential phase, but in the reverse order. In the stationary phase, the relative content of palmitic acid decreased even further in BB-46 (*P* ≤ 0.05). Instead, nonanoic acid (C9:0) became the second most dominant fatty acid after C18:1 in all 3 biological replicates (Fig. 6). Moreover, the mean percentage of stearic acid (C18:0) decreased significantly in the stationary growth phase in BB-46 (*P* ≤ 0.05) (Fig. 6). In contrast, significantly increased mean percentages of palmitic, oleic, and myristic acid (C14:0) (*P* ≤ 0.05) were observed in BB-12 (Fig. 6).

Fatty acid analysis further revealed a significantly higher content of palmitic acid in both growth phases in BB-12 than in BB-46 (*P*_adj_ ≤ 0.05) (Fig. 6 and Table S3). Furthermore, significantly lower mean percentage of oleic acid and mean average ratio of unsaturated to saturated fatty acids (UFA/SFA) were detected in BB-12’s cell membrane in both growth phases (*P* < 0.05) (Fig. 6). In contrast, no difference in the mean length of the fatty acids was observed.

**FIG 7:**
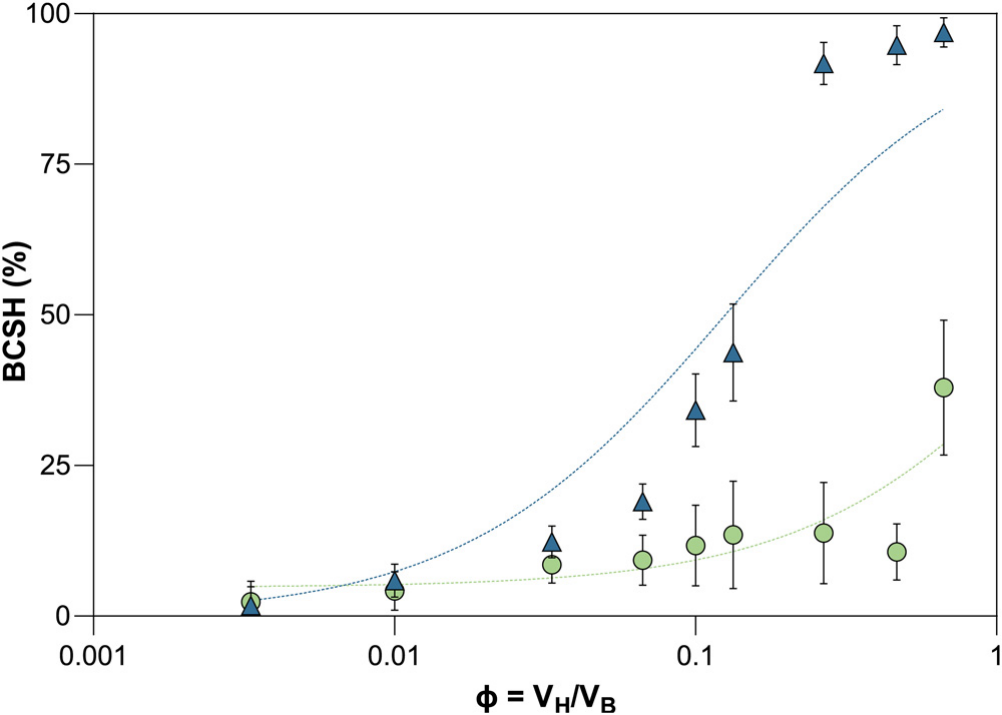
Interfacial adhesion curves of *B. animalis* BB-12 and B. longum BB-46 in 100 mM sodium phosphate buffer at pH = 7. Bacterial cell surface hydrophobicity (BCSH) of BB-12 (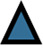) and BB-46 (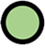) over the volume ratio (ϕ) of the hexadecane. The volume ratio is calculated as VH/VB, where VH and VB are the volumes of the hexadecane and the buffer phase for each sample, respectively. Each sample point represents the mean of a technical triplicate ± standard deviation. The interfacial adhesion curves were fitted to the data using a previously published equation (59).

As a second characteristic of the cell envelope, the bacterial cell surface hydrophobicity (BCSH) (41) was assessed in the stationary phase. With increasing volume ratio of hexadecane to cell suspension (ϕ), the relative BCSH value determined for BB-46 increased only slightly. In contrast, the BSCH value clearly increased for BB-12. At the maximum ϕ of 0.67, BB-12 reached adhesion of 97% ± 1% to hexadecane, while the adhesion of BB-46 at the maximum ϕ tested was 38% ± 11% (Fig. 7).

### Polyphosphate accumulation.

The accumulation of polyphosphate in bacteria, including bifidobacteria, can be triggered by exposure to various stressors, and improve their stress resistance (41–44). However, the capability to accumulate polyphosphate differs among species (45), and depends on the cultivation conditions (41, 43, 46). Using epifluorescence microscopy, putative polyphosphate granules were detected at the cell poles of both BB-12 and BB-46 when cultivated in CDM. No clear differences could be detected between the strains (Fig. S2).

## DISCUSSION

### Strain-specific robustness and stability.

The higher survivability of BB-12 under most applied storage conditions implies that the strain has overall higher robustness and stability than BB-46. This is in agreement with previous studies, reporting exceptionally high robustness and stability for BB-12 (7, 8) but high sensitivity for B. longum strains toward stress (7, 8). Overall, the results suggest that BB-12 is equipped with defense mechanisms that allow it to cope with various stressors, and that are not present in BB-46.

### Growth dynamics and metabolite profile.

In BB-12, lactate production seemed to be the main route for NAD^+^ regeneration, whereas the low lactate and relatively high formate formation in BB-46 indicated that NAD^+^ regeneration was coupled to ethanol formation in the latter. Ethanol formation by BB-46 in CDM has been evidenced before in crimp-top serum bottles (18). However, in this study, ethanol measurements were inconclusive, possibly because of the continuous gas stripping of the bioreactor headspace.

The production of individual amino acids by *Bifidobacterium* strains, as observed for BB-12 and BB-46 in the present study, has been reported before (47, 48). In line with our findings, l-alanine, l-valine and L-aspartate have been found to be the most commonly secreted amino acids by *Bifidobacterium* strains (47). The physiological significance of amino acid secretion by bifidobacteria has not been investigated. In general, amino acid secretion can be a consequence of overflow metabolism or hypoosmotic stress (49).

As the strains were kept in their fermentation broth during storage, it cannot be excluded that differences in the production and consumption of metabolites influenced their survivability when harvested in the stationary phase. However, the medium composition in the exponential phase was comparable between strains.

The reason behind the decelerated growth of BB-46 once reaching an OD_600_ of 1.3 could not be verified. One plausible explanation is the depletion of a growth rate limiting but non-essential nutrient. Exposure to such nutrient limitation might affect the phenotype, including the robustness and stability of the strain.

### Strain-specific transcription of stress-associated genes.

We recently demonstrated that *Bifidobacterium* strains, including BB-12 and BB-46, harbor different sets of stress-associated genes (20). For example, in contrast to BB-46, BB-12 harbors genes encoding formyl-CoA transferase and oxalyl-CoA carboxylase (20). Their high transcription level in BB-12 most likely contributes to the strain’s probiotic effect in the human gastrointestinal tract as, well as to its oxalate and acid-resistance (26).

In addition, the higher transcription levels of several stress-associated genes, e.g., oxidative and osmotic stress-associated genes, in BB-12 than in BB-46 suggests that BB-12 is intrinsically equipped for exposure to various stressors. This might allow for an instant response and enhanced survivability of the strain, compared to BB-46. Likewise, a previous study reported constantly high expression of BSH in BB-12 on the transcriptional and translational level, and suggested a selective advantage due to the ability to immediately respond to bile salts (50). In this study, the high expression of oxidative stress-associated genes might contribute to the strain’s survivability under the applied aerobic storage conditions. For example, the BCP-type peroxiredoxin might combat lipid peroxidation under aerobic conditions, as has been suggested for BCP in E. coli and *Heliobacter pylori* (51, 52). Moreover, relatively high expression of genes encoding mechanosensitive channels and a potassium transporter in BB-12 might be associated with an enhanced resistance to changing osmolality by allowing the maintenance of the turgor pressure upon osmotic stress. Finally, the significantly higher transcription level of heat shock proteins in BB-12 compared to BB-46 is likely associated with enhanced maintenance of protein folding upon exposure to stressors. It can be hypothesized that the robustness and stability of BB-46 could be improved by triggering the strain’s expression of stress-associated genes that were found to be highly expressed in BB-12.

### Protective effect of l-methionine in BB-12.

Under aerobic conditions, ROS can damage proteins by oxidation of amino acid residues, with the 2 sulfur-containing amino acids methionine and cysteine being most susceptible to oxidation (24). The activity of MsrA and MsrB is linked to a ROS-scavenging l-methionine oxidation-reduction cycle (24). Commonly, MsrA and MsrB exist as individual enzymes; however, in some bacteria, they are fused in a single protein MsrAB, as described previously in Streptococcus pneumoniae (53). Likewise, Msr encoded in the genome of BB-12 and BB-46 belongs to the MsrAB type.

The large uptake of l-methionine by BB-12 during growth (Fig. 2A) and the upregulation of multiple genes linked to the biosynthesis of l-methionine in the stationary phase might ensure a high intracellular l-methionine concentration in BB-12 to promote the ROS-scavenging l-methionine oxidation-reduction cycle. This should counteract the detrimental effect of ROS on protein homeostasis and free amino acid supply, and thereby contribute to the strain’s good survival under aerobic conditions.

The hypothesis of increased l-methionine biosynthesis from L-homoserine is challenged by the observation that the genes linked to the supply of L-homoserine from L-aspartate were downregulated in the stationary phase in BB-12. However, as 2 of the enzymes are reversible, the downregulation might aim to limit the reverse reaction from L-homoserine to L-aspartyl-4-phosphate. Moreover, while L-aspartate was secreted by BB-12 during growth, small amounts were taken up in the stationary phase, which might fuel l-methionine biosynthesis.

In the stationary phase, the uptake rate of l-methionine in BB-12 decreased at a concentration of 0.04 mM, even though the transcription of four homologs of genes of the *metNPQ* operon (18, 23) was upregulated. This might be due to a low substrate affinity of the transporters, as well as a lack of ATP. Moreover, 3 of the upregulated genes encode subunits of a single ABC-type transporter in BB-12, while the 4^th^ gene codes for the substrate-binding protein of a second ABC-type transporter that was stronger expressed than the other transporter in the exponential phase. The second transporter might rather facilitate the active uptake of l-methionine sulfoxide, and thereby fuel the ROS-scavenging cycle of MsrAB in BB-12 under aerobic conditions.

Finally, the activity of the MsrAB system depends on the regeneration of reduced MsrAB by TrxR at the expense of NADPH (54). In line with this, elevated transcription of genes encoding TrxR and TrxA were observed in BB-12 in the stationary phase. However, how the strain ensures sufficient supply of NADPH for the system, as well as for other NAD(P)H-dependent ROS-detoxification systems, in the stationary phase remains to be understood.

In contrast to BB-12, the ROS-detoxification mechanism linked to MsrAB activity appears not to be active in BB-46 under the given conditions, since (i) almost no l-methionine was taken by BB-46, (ii) the strain showed a relatively low expression level of the *msrAB* gene, and (iii) genes associated with l-methionine biosynthesis were not upregulated in the stationary phase. This might partly explain the strain’s poorer robustness and stability of BB-46 under aerobic conditions, both in the exponential phase, as well as when stored for an extended time of 28 days. Overall, these results suggest that the addition of l-methionine to the medium might promote the robustness and stability of some *Bifidobacterium* strains.

### Bacterial cell surface hydrophobicity.

It is conceivable that the high hydrophobicity of BB-12 may contribute to enhancing its survivability during short-term storage. To date, no clear correlation between increased BCSH and enhanced stress resistance could be established in bifidobacteria, but previous studies have shown that BCSH changes along with stress tolerance acquisition or in response to stress exposure (9, 10, 55). In this study, the strong hydrophobic surface of BB-12 was a likely cause of its sedimentation during storage by promoting auto-aggregation in the aqueous culture broth (56, 57). This in turn might minimize the exposure of BB-12 cells to environmental stressors, such as oxygen, during storage. Such increased stress tolerance due to autoaggregation, manifested as improved tolerance against lignocellulose-derived metabolic inhibitors, has been proven in yeast (58).

In general, the BCSH is determined by the polarity of molecules exposed on the cell surface or located within the cell wall, such as cell-wall associated polysaccharides, (lipo-) teichoic acids, and cell surface-associated proteins (59). The different BCSH of BB-12 and BB-46, thus, implies that they differed in their cell surface decoration under the given cultivation condition. The low initial plateau of the interfacial adhesion curve of BB-12 (Fig. 7) suggests that the hydrophobic moieties of its cell wall are in its inner part, capturing small quantities of hexadecane without disturbing the cell surface characteristics, and causing extraction from the aqueous solution (59). One contribution to the higher BCSH of BB-12 than of BB-46 might be a greater abundance of the hydrophobic portion of lipoteichoic acids (LTA), previously reported to contribute to BCSH in bifidobacteria (60), on the cell surface of BB-12. However, this hypothesis conflicts with the higher expression level of a gene encoding an LTA synthase family protein in BB-46 [log_2_(FC)_BB12EXP/BB-46EXP_: −4.0 and log_2_(FC)_BB12STAT/BB-STAT_: −3.5] (Data set S4 and 5).

Another explanation could be a higher abundance of hydrophobic surface (S)-layer proteins on the cell surface of BB-12. In general, S-layer proteins can form a protective coating around the peptidoglycan layer in Gram-positive bacteria (61), but very little is known about S-layer proteins in bifidobacteria (62). According to genome annotation, BB-12 possesses 2 S-layer homology domain-containing proteins (BIF_01636, BIF_01633). In addition, BB-46 harbors 1 gene annotated as ABC transporter permease (I3242_02465) that shows 99% sequence identity with an S-layer protein of B. longum DJO10A (62). These S-layer proteins of BB-12 and BB-46 have a similar percentage of hydrophobic amino acids (40 to 42%). In BB-46, the genes encoding the putative S-layer protein and a polysaccharide pyruvyl transferase family protein, found to be involved in the binding of proteins with S-layer protein homology domain in Bacillus anthracis (63), were significantly downregulated in the stationary phase [log_2_(FC): −2.8]. Thus, a decreased S-layer protein biosynthesis might affect the surface characteristics of BB-46 in the stationary phase. In contrast, the expression level of the genes encoding the putative S-layer proteins in BB-12 did not change significantly between growth phases.

Besides S-layer proteins, surface-associated moonlight proteins might affect the hydrophobicity of the 2 strains. For example, BSH has been evidenced in the cell-wall fraction of *B. animalis* BI07, and was suggested to be exposed on the cell surface to function as a plasminogen-binding protein (64). The highly expressed BSH in BB-12 might also be exposed on the cell surface, and, thus, determine its BCSH. The hydrophilic surface of BB-46 might further indicate that BB-46 possessed more non-hydrophobic polysaccharides on its surface in the stationary phase than BB-12 did (59).

Taken together, comparing the cell wall composition of the 2 strains may identify the cellular components underlying BB-12’s high hydrophobicity and BB-46’s relative hydrophilicity. However, the correlation between BCSH and robustness and stability in bifidobacteria needs to be further studied.

### Fatty acid profile.

One important characteristic of the cell membrane composition is the UFA/SFA ratio, which has been shown to influence the fluidity and permeability of the membrane (65). In contrast to a previous study on the effect of the growth phase of B. longum R0175 on its fatty acid profile (39), no significant change in the UFA/SFA ratio was observed for BB-12 and BB-46 between the exponential and stationary phase. However, our data suggests a lower UFA/SFA ratio in the cell membrane of BB-12 than in that of BB-46 (Fig. 6). A lower relative content of unsaturated fatty acids in the cell membrane of BB-12 may be associated with a higher membrane stiffness and lower membrane permeability (65). A lower UFA/SFA ratio and/or a reduced membrane fluidity has been proposed to contribute to increased freezing resistance in B. longum R0175 (39), as well as higher acid-resistance of derivatives of B. longum JDY1017 and B. breve BB8 (38). Moreover, a decrease of the UFA content has been reported for the bile-resistant derivative of *B. animalis* 4549dOx compared to its parental strain under non-stressed condition (66). Our study provides additional support for a positive correlation between a low UFA/SFA ratio of the cell membrane of *Bifidobacterium* strains and high stability and robustness.

Because changes in the fatty acid composition of BB-46’s membrane in the stationary phase resulted in a profile that was even less similar to that of BB-12, it seems unlikely that these alterations contributed to the strain’s increased survivability in the stationary phase. The change in the fatty acid profiles of both strains in the stationary phase might be associated with the downregulation of long-chain fatty acid–CoA ligases, which are responsible for the incorporation of exogenous fatty acids into the cell membrane (14, 40).

In agreement with the difference between BB-12 and BB-46, lower relative C16:0 and C14:0 contents, as well as higher C18:1 content, have previously been detected in the cell membrane of *B. lactis* DSM 10140 compared to *B. lactis* BL-04 (14). Despite having hardly any genetic differences to *B. lactis* DSM 10140 (67), *B. lactis* BL-04 showed a higher intrinsic resistance to H_2_O_2_ (5). However, a higher C19:0 cyclopropyl plasmalogen content in the cell membrane of *B. lactis* BL-04 than in the cell membrane of *B. lactis* DSM 10140 has been speculated to be decisive for its higher intrinsic resistance to H_2_O_2_ (68). Cyclopropanation might protect the cell membrane against lipid peroxidation by stabilizing the unsaturated bonds through addition of a methyl group (14). In this work, the cyclopropane fatty acid content in the cell membrane of the 2 strains was not determined, but differential gene expression analysis revealed a higher expression of cyclopropyl-fatty-acyl phospholipid synthase in BB-12 than in BB-46 across growth phases (14).

### Growth phase-dependent stress tolerance mechanism.

In agreement with our results, previous studies showed no effect of the harvesting time of BB-12 (= E-012010) on the strain’s freeze-drying resistance (69), whereas B. longum R0175 displayed a higher survival rate during freezing when harvested in the stationary phase (39). Thus, the growth phase-dependent stress tolerance may be a general characteristic for B. longum strains. When entering the stationary phase, bacteria are known to reprogram their gene expression in order to ensure survival, often including the upregulation of genes linked to improved stress resistance (70).

### Response to carbon starvation.

The increased transcription of genes linked to the uptake and utilization of various carbohydrates in BB-46 and, especially, in BB-12 when entering the stationary phase, agree with a recent study showing enhanced transcription of genes linked to carbohydrate metabolism by *B. animalis* subsp. *lactis* A6 in the stationary phase (71). Together, these findings indicate a high degree of catabolic flexibility of *Bifidobacterium* strains, as well as an intrinsic ability to cope with variations in substrate availability and adapt to changing conditions in their natural habitat. The simultaneous downregulation of genes associated with the bifid shunt spares the cells from spending energy on superfluous enzyme production. Furthermore, the upregulation of genes encoding nonspecific nucleoside hydrolases in the stationary phase, might provide BB-12 and BB-46 with ribose from the hydrolysis of purine and pyrimidine nucleosides, as suggested for Lactobacillus plantarum 423 under acid stress condition (72). Ribose may be used as a carbon source to meet minimal energy requirements for survival under carbon starvation. This hypothesis is in line with the upregulation of genes encoding ribokinase in both strains in the stationary phase.

The reason for the strong upregulation of a gene annotated as acetoin reductase in BB-12 in the stationary phase remains unclear, but it is conceivable that in its natural habitat, it might allow NAD^+^ regeneration using diacetyl secreted by intestinal lactic acid bacteria (73).

### Enhanced robustness and stability of BB-46 in the stationary phase.

Some of the described growth phase specific expression profile in BB-46 might explain the strain’s better survivability when harvested in the stationary rather than in the exponential phase: (i) First, the increased transcription of the BfeUO transport system in BB-46 might enhance iron uptake in the stationary phase, and thereby contribute to maintenance of iron homeostasis upon oxidative stress during the short-term storage. (ii) The upregulation of a gene encoding a cold shock protein, which might function as RNA chaperone (74), may contribute to BB-46’s better survival in the stationary phase. (iii) A reduced expression of the *npox* homolog in the stationary phase compared to the exponential phase might be associated with less H_2_O_2_ being formed by NPOX activity under the aerobic storage condition. Even though the expression of the H_2_O_2_-reducing AhpC-TrxR system is high in the exponential phase, the efficiency of the system might be too low in BB-46 to avoid H_2_O_2_ accumulation, as previously suggested for other O_2_-sensitive *Bifidobacterium* strains (75). In contrast to BB-46, the relatively higher expression of the *npox* homolog in BB-12 might not have a detrimental influence on the strain’s survival under aerobic conditions due to the generally high expression of ROS-detoxifying enzymes in BB-12. In general, the reasons for the high expression of multiple oxidative stress-associated genes by BB-46 during growth and their significant downregulation in the stationary phase under anaerobic cultivation conditions are not clear. It would rather be expected that these genes were upregulated in the stationary phase as part of a general stress response to nutrient limitation. (iv) The upregulation of multiple genes linked to DNA repair in the stationary phase might be associated with enhanced maintenance of genomic DNA integrity by BB-46 during storage. Based on a previous study, a loss of genomic DNA integrity was suspected to cause the loss of culturability in probiotic strains during storage in dry state, suggesting that the effectiveness of a strain’s DNA protection and repair plays a crucial role in the maintenance of viability (76). (v) Finally, the elevated transcription of 4 genes associated with fatty acid synthesis might improve the maintenance of BB-46’s membrane integrity during storage due to improved availability of fatty acid components. In line with this hypothesis, a decrease in the expression of FAS and BioY has previously been observed in B. breve UCC2003 and B. breve DSM 20213 when exposed to bile and linoleic acid stress, respectively, and was suspected to be associated with reduced membrane integrity under stress conditions (77, 78).

In BB-46, the upregulation of genes encoding bile transporters is likely part of the strain’s general stress response as no bile was added to the medium. The consequences of the upregulation of transposase genes in BB-46 in the stationary phase on its survivability during short-term storage are unknown, but transposase activity might present an evolutionary advantage upon environmental change, as suggested before for Lactobacillus casei ATCC 334 (79).

### pH-dependent viability of BB-46.

The high susceptibility to acidic storage conditions of BB-46 might be partly explained by the low expression level of most genes encoding subunits of F_1_F_0_-ATPase in BB-46 in the stationary phase, resulting in a loss of pH homeostasis under acidic conditions. A higher expression level of the corresponding genes during exponential growth might be explained by the sucrose transport mechanism used by BB-46. Based on gene expression levels, sucrose was mainly taken up by a H^+^-symporter in BB-46, which might require a high H^+^-translocating F_1_F_0_-ATPase activity. In contrast, sucrose appeared to be taken up by a sodium symporter in BB-12.

The discrepancy between active cell count using flow cytometry and the viable cell counts for BB-46 when stored at low pH, as well as for 28 days at neutral pH, implies that a considerable fraction of BB-46 cells retained their cell membrane potential despite losing their ability to replicate. It can be speculated that these cells entered an active but nonculturable (ABNC), or active but ultimately culturable (dormant), state (80). This is in line with the findings of previous studies that investigated the survivability of B. longum BL46 when stored in fermented milk; the majority of cells maintained their membrane integrity, esterase activity, intact pH gradient, and 16S rRNA levels, even though the viable cell count decreased drastically (81, 82). To date, it has not been evidenced that *Bifidobacterium* strains can regain their ability to replicate once having entered the ABNC state, and the implications of potentially entering such a state on their probiotic application remains to be investigated.

### Conclusions.

In this study, we observed considerable differences in physiological and metabolic characteristics in lab-scale batch cultivations of BB-12 and BB-46; 2 industrially and clinically relevant probiotic strains exhibiting different degrees of stress tolerance. In BB-46, the degree of stress resistance appears to be dependent on the growth phase. Even though BB-46’s stress resistance improves in the stationary phase, the survival of BB-12 remains superior under acidic conditions or extended storage periods.

Several physiological and metabolic characteristics of BB-12 and BB-46 may contribute to the differences in their stress physiology. First, the 2 strains differed in growth and fermentation product profiles, as well as amino acid utilization profiles in batch cultivations. We suggest that a higher uptake of l-methionine by BB-12 in the exponential phase contributes to a stronger ROS-detoxifying MsrAB activity during storage under aerobic conditions, compared to BB-46. Moreover, the upregulation of l-methionine biosynthesis in BB-12 in the stationary phase might also promote MsrAB activity.

Differential gene expression analysis revealed constantly high expression levels of several stress-associated genes in BB-12, such as ROS-detoxifying enzymes, bile salt hydrolase, and chaperones. This should allow the strain to immediately combat environmental stressors once they are encountered, which, in turn, might enhance its survivability. The high viability loss of BB-46 under acidic conditions might be due to a low expression level of genes encoding the F_1_F_0_ ATPase. In addition, the improved stability of BB-46 in the stationary phase, compared to the exponential phase, is likely a result of changed gene expression, e.g., the upregulation of genes associated with DNA repair and fatty acid metabolism.

Differences in the cell envelope characteristics of the strains also appear to contribute to their different stress physiology. While the cell surface of BB-12 was strongly hydrophobic, the BB-46 cell surface was relatively hydrophilic. In addition, a significantly higher content of C16:0 fatty acids and a lower content of C18:1, together with an overall lower UFA/SFA ratio in the cell membrane of BB-12, might contribute to the strain’s enhanced robustness and stability compared to BB-46. In contrast, polyphosphate granules were detected in the cytoplasm of both strains, which suggests that polyphosphate formation does not seem to determine their deviating stress resistance.

Based on the presented comparison of BB-12 and BB-46, it is likely that the variation in robustness and stability of the 2 strains is a result of the combination of metabolic and physiological differences, rather than of a single factor. However, the specific features that contribute to stability and robustness in individual bifidobacterial strains remain to be validated in future studies.

The results presented herein indicate important genomic and physiological contributions to robustness and stability of bifidobacteria. The results and applied methodologies can facilitate the selection of novel, commercializable strains, and guide the optimization of production processes for improved robustness and stability of strains with promising health benefits.

## MATERIALS AND METHODS

### Bacterial strains.

The strains *B. animalis* subsp. *lactis* BB-12 and B. longum subsp. *longum* BB-46 were obtained from the Chr. Hansen Culture Collection. Strains were maintained in in 20% (vol/vol) glycerol at −80°C.

### Preculture procedures and strain maintenance.

Strains were cultivated anaerobically by 2 consecutive subcultures in 50 mL CDM (18) with 10 g L^−1^ sucrose. The cultivations were performed in 100-mL crimp-top serum bottles as described before (18).

### Batch fermentations.

Strains were cultivated in 200 mL CDM with 10 g L^−1^ sucrose in pH-controlled batch fermentations in a 250-mL DASGIP Parallel Bioreactor System, controlled using Dasware Control version 5.5.1 (Eppendorf). Cysteine-HCl and menaquinone-4 were added prior to inoculation at final concentrations of 0.5 g L^−1^ and 0.001 g L^−1^, respectively. Precultured cells were harvested by centrifugation at 8,000 rpm and 4°C for 4 min and used for inoculation to an initial OD_600_ of 0.05. The medium was cold-inoculated at 5°C. To initiate the fermentation, the temperature was increased to 37°C at a specific time, selected based on the lag phase duration of each strain. During cultivation the temperature was maintained at 37°C. The pH was maintained at 6.5 through automatic titration with 13.5% NaOH. To maintain anaerobic conditions, the headspace was continuously flushed with 80% N_2_ and 20% CO_2_ at a flow rate of 3 L h^−1^. Agitation was set to 250 rpm using one Rushton impeller and one pitched-blade impeller.

### Assessment of viability during short-term storage.

The stress tolerance of the strains was assessed by testing their survival during short-term storage under suboptimal conditions. Cells were collected from each replicate in the exponential and stationary phases. All samples were stored away from light at 8 to 10°C. Cells harvested in the exponential phase were stored for 7 days in the culture broth (pH 6.5), whereas cells harvested at the stationary phase were additionally kept for 28 days at pH 6.5, and for 7 days at pH 5.5 and pH 4.5. The pH in the samples were adjusted using HCl.

**(i) Viable cell count.** The number of viable cells in the culture broth was determined before and after storage in triplicates by CFU, applying the pour plate technique. The cell suspension was vortexed for 1 min at 24,000 rpm to remove cell aggregation. After decimal dilutions in saline-peptone solution (8.5 g L^−1^ NaCl, 1 g L^−1^ peptone, pH 7), either 0.5 mL or 1.0 mL of the suspension was plated on MRS agar (Difco, pH 6.5) containing 0.5 g L^−1^ cysteine-HCl. The temperature of the molten agar medium was 46 ± 1°C. The plates were incubated for 48 h to 72 h at 37°C in an anaerobic jar in an atmosphere containing < 0.1% O_2_ and 7 to 15% CO_2_ (Oxoid, AN0035A). The viable cell (CFU) loss during storage of the strains was calculated in log_10_ units (log_10_ loss) as an indicator for their robustness and stability.

**(ii) Flow cytometry.** Flow cytometry was applied to determine the number of active cells before and after storage using the dye 3,3′-diethyloxacarbocyanine iodide (DiOC), which is sensitive to membrane potentials via a red-shifted fluorescence (83). Samples were stored in 20% glycerol (vol/vol) at −80°C until analysis. Just before analysis, the cells were reactivated in MRS medium (20 g L^−1^ glucose, pH 6.5) supplemented with 0.5 g L^−1^ cysteine-HCl for 30 min at 40°C. The samples were analyzed with a LSR II Flow cytometer (Becton, Dickinson) set up with dual threshold on forward and side scatter, and equipped with a 488 nm blue laser and a 650LP filter, detecting the far-red fluorescence. The green light was measured at the emitted wavelength of 515 to 545 nm. Cells and standard beads were detected in a plot of forward versus side scatter and gated into plot of red versus green fluorescence to differentiate between metabolically active and inactive cells (83). One part of activated cells was stained with 24 parts of dye solution for 30 min at 20°C. The dye solution consisted of 3 μM DiOC, 0.2% glucose (wt/wt), 130 mM NaCl, 3 mM NaH_2_PO_4_-H_2_O, 7 mM Na_2_HPO_4_-12 H_2_O, 0.05% Tween80, and 0.01% (vol/vol) of Yellow-green Fluospheres (around 4.5 × 10^5^ beads mL^−1^). The injection flow rate was 120 μL min^−1^. The correct detection of cells with intact membrane potential was verified by controlled inactivation using 15 μM of the protonophore carbonyl cyanide chlorophenylhydrazone (CCCP) to depolarize the cells. The analysis was run in duplicates. The cell detection limit of the total cell counts was 10^8^ cells mL^−1^.

### Fatty acid analysis.

The relative fatty acid composition of cellular lipids was determined on a gas chromatography – mass spectrometry (GC-MS) system (ISQ LT, Thermo Scientific) equipped with a ZB-FAME column (20 m x 0.18 mm x 0.15 μm, Phenomenex). Samples of 15 mL cell culture were harvested in the early exponential and stationary phase by centrifugation at 15,557 ×g for 10 min at 5°C, washed with 5 mL phosphate-buffered saline ([PBS], Sigma-Aldrich, pH = 7.2 to 7.6) and frozen at −20°C until analysis. The analysis was performed by Chalmers Mass Spectrometry Infrastructure at Chalmers University of Technology, Gothenburg, Sweden. Prior to analysis, 25 μg of C17:0 internal standard (5 mg mL^−1^), 1 mL of 10% (vol/vol) methanol-HCl solution, and 1 mL toluene were added to the approximately 50 mg cell pellet, vortexed 5 min at 1600 rpm and incubated at 70°C for 20 h. The resulting fatty acid methyl esters (FAMEs) were extracted in 2 mL n-hexadecane. Phases were separated by centrifugation for 3 min at 3000 rpm and 1 mL of the upper organic phase was analyzed in GC-MS. GLC-463 (Nu-Chek Prep) was used as reference standard. The statistical difference between means was tested applying a multiple paired (across growth phases for a given strain) and unpaired (across strains for a given growth phase, Welch correction) *t* test (Holm-Šídák method), respectively, using GraphPad Prism version 9.3.1 (GraphPad Software). The *P* value for single testing and the multiplicity adjusted *P* value (*P*_adj_) were obtained.

### Growth profiling.

The optical density of the culture broth at 600 nm (OD_600_) was determined using a Genesys 10S UV-Vis spectrophotometer (Thermo Scientific). The final cell dry weight (CDW) of each cultivation was quantified by filtering 3 mL of cell suspension through pre-dried membrane filters (0.22 μm), washing three times with equivalent volumes of Milli-Q water, and drying to constant weight.

### Metabolite analysis.

Sucrose concentrations were determined by HPAEC-PAD as previously described (18). To arrest metabolic conversion, 2.00 mL ice-cold 96% (vol/vol) ethanol were added to 2.00 mL cell broth.

The concentrations of fermentation products were determined by HPLC (Ultimate 3000, Thermo Fisher Scientific) using a Rezex ROA H+ (8%) ion-exclusion column (Phenomenex), and both UV (210 nm) and RI detectors at 65°C with a mobile phase of 5 mM H_2_SO_4_ and a flow rate of 0.6 mL min^−1^.

The concentrations of glutamine and arginine were determined on a LC-MS system (Acquity UPLC and Xevo TQ-XS, Waters Corporation) using an AccQ-Tag Ultra RP column (130 Å, 1.7 μm, 2.1 mm x 100 mm, Waters Corporation). The samples were centrifuged at 4°C at 15,000 × *g* for 5 min, and the supernatant was diluted 60 times in Milli-Q water. The amino acid derivatization was performed using the AccQ-Tag Ultra Derivatization Kit (Waters Corporation). The sample was mixed 1:1 with an internal standard solution containing 25 μM all the targets labeled with ^13^C. The mix was buffered using 50 μL of borate buffer, and 20 μL of the derivatization reagent was added. The solution was mixed at 2000 rpm and 55°C for 10 min before being analyzed on the LC-MS.

The concentrations of all other amino acids were measured on a GC-MS system (7890A and A5975, Agilent) using a DB-XLB column (Agilent) and helium as the carrier gas. The samples were diluted 1:1 with 1% (wt/vol) ascorbic acid and centrifuged at 4°C at 15000 × *g* for 5 min. A total of 25 μL of the supernatants were diluted with 225 μL Milli-Q water and supplemented with 50 μL of 1 mM L-norvaline as internal standard. For the amino acid derivatization, 200 μL of a 4:1 (vol/vol) methanol/pyridine mixture, 25 μL methyl chloroformate, and 500 μL of 1% (vol/vol) methyl chloroformate/chloroform was added to the samples. After phase separation at 4°C, the organic phases were analyzed on the GC-MS.

### Gene expression analysis using RNA-Seq.

**(i) RNA extraction.** Cell broth samples for RNA-seq were immediately treated with RNAprotect Bacteria Reagent (Qiagen), aliquoted in 1 to 2 mL, centrifuged for 2 min at 10000 rpm, and stored at −80°C. The RNA was extracted from the samples using the RNeasy minikit (74104, Qiagen), according to the manufacture’s instruction with the following modifications: Enzymatic lysis of a cell pellet was performed by resuspending the cells in 200 μL TE buffer containing 15 to 30 mg mL^−1^ lysozyme, 15 μL Proteinase K (20 mg mL^−1^, Qiagen) and 2 to 4 μL mutanolysin (25 U μL^−1^), and incubation for 10–30 min at 37°C and 3 × g in a Thermomixer. Residual DNA was eliminated by column digestion with RNase-free DNase I (Qiagen) for 15 min at 25°C. RNA was eluted in a RNase-free tube in 110 μL of RNase-free water. RNA integrity was assayed using an Agilent 2100 Bioanalyzer G2938B (Agilent Technologies) and quantified using a Qubit 3.0 Fluorometer (Life Technologies).

**(ii) RNA sequencing.** RNA-Seq library preparation and sequencing were performed by GENEWIZ. RNA-Seq libraries were prepared using NEBNext rRNA Depletion Kit (Bacteria) for rRNA depletion and NEBNext Ultra II Directional RNA Library Prep Kit for Illumina, following manufacturer’s instructions (NEB). Sequencing libraries were validated using DNA Kit on the Agilent 5600 Fragment Analyzer (Agilent Technologies), and quantified using Qubit 4.0 Fluorometer (Invitrogen). The libraries were sequenced using a NovaSeq 6000 system (Illumina Inc.) using a 2 × 150 Pair-End (PE) configuration. Image analysis and base calling were conducted by the NovaSeq Control Software v1.6.

**(iii) RNA-Seq data processing.** Sequencing reads were trimmed using Trimmomatic 0.39, mapped using BBMap 38.90.0, and read counts were calculated using htseq 0.13.5. Differential gene expression analysis was performed by fitting generalized linear models with negative binomial distributions using the DESeq2 1.30.0 R package (84). The statistical significance of the differential expression was assessed using Wald tests for each gene. The *P* values of each gene were adjusted for false discovery rate using the Benjamini–Hochberg method (85). Gene expression of each strain was compared between the exponential and stationary growth phases. Additionally, gene expression and regulation of orthologous genes in BB-12 and BB-46 were compared. A gene was considered to be differentially expressed if the adjusted *P* value (*P*_adj_) ≤ 0.01 (false discovery rate) and the absolute value of log_2_foldchange was greater than 2 [|log_2_(FC)| ≥ 2]. To search for biologically significant patterns in the transcriptome data, Pathway Tool Omics Dashboard (86) was used for visualization and categorization of differentially expressed genes in functional groups, importing only log_2_(FC) values with an adjusted *P* value ≤ 0.01. Orthologs in BB-12 and BB-46 were identified using Proteinortho 6.0.28 with default settings (87). Significantly, differentially expressed genes were classified into Clusters of Orthologous Groups of proteins (COGs) (88) using the online service eggNOG-mapper 2.1.7 (89).

Genes in the genome sequences of BB-12 (Accession no. CP001853.2) and BB-46 (Accession no. CP065209) were functionally annotated with NCBIPGAP (90). Moreover, knowledge from a previous homology search of stress-associated genes across *Bifidobacterium* strains (20), as well as from the genome-scale metabolic models of BB-12 (*i*AZ480) and BB-46 (*i*MS520) (18), was used to assign more precise functions to differentially expressed genes.

### Hydrophobicity measurement.

The BCSH was assessed using microbial adhesion to hydrocarbons (MATH) (91) in combination with interfacial adhesion curves (59). Cells were harvested in the stationary phase at 5,000 rpm at 5°C for 20 min and stored at −20°C until analysis. The cell pellet was washed three times in 10 mL 100 mM sodium phosphate buffer (pH 7), and centrifuged at 5,000 rpm at 5°C for 20 min. After resuspension in buffer to OD_600_ 0.5 ± 0.05, the cell suspension was divided into 3 mL aliquots. To obtain a series of BCSH values, varying amounts of hexadecane were added (10 μL to 2000 μL) to the cell suspension, the mixtures were vortexed twice for 30 s to 60 s at the highest speed, and then rested until complete phase separation. Each volume ratio (φ) of the hexadecane to cell suspension was prepared in triplicates. The BCSH was calculated as follows: {[1 − OD_600_ (aqueous phase)]/OD_600_ (initial)} · 100. The average BCSH values were plotted against the φ, and the interfacial adhesion curves were fitted to the data based on the equation published by Schär-Zammaretti and Ubbink (59).

### Polyphosphate detection.

To assess for polyphosphate accumulation, the strains were cultivated in CDM in crimp-top serum bottles (see section 6.2) and harvested in the exponential phase. Cells were fixed with 4% formaldehyde for 30 min at room temperature, washed twice with PBS buffer (pH 7.2 to 7.6), and stored at 4°C overnight. Before analysis, the cells were permeabilized with 0.3% Triton X-100 for 10 min. Subsequently, the cells were incubated with 50 μg mL^−1^ 4,6-diamidino-2-phenylindole (DAPI) for 10 min. The samples were analyzed using an Eclipse Ti2 epifluorescence microscope (Nikon). DNA was visualized by excitation at 365 nm and emission at 460 nm. Polyphosphate granules were visualized by excitation at 469 nm and emission at 525 nm.

### Ethical approval and consent to participate.

Not applicable.

### Data availability.

We declare that all the data supporting the work are available within the paper and its Supplementary Information.
