# Supplementary material for: Novel Insights into the Molecular Mechanisms Underlying Robustness and Stability in Probiotic Bifidobacteria
Source: Appl Environ Microbiol. 2023 Feb 21;89(3):e00082-23. doi: 10.1128/aem.00082-23 (PMC10057886; doi:10.1128/aem.00082-23)
Supplement: Supplemental file 1 — Supplemental material. Download aem.00082-23-s0001.pdf, PDF file, 0.4 MB [file aem.00082-23-s0001.pdf]

Supplementary material - Metabolic and physiological differences of two *Bifidobacterium* strains differing in robustness and stability

Marie Schöpping[1,3], Anisha Goel[2], Kristian Jensen[1], Carl Johan Franzén[3], Ahmad A. Zeidan[1]

[1]Systems Biology, Discovery, Chr. Hansen A/S, 2970 Hørsholm, Denmark

[2] Process Upscaling, Chr. Hansen A/S, 2970 Hørsholm, Denmark

[3] Division of Industrial Biotechnology, Department of Biology and Biological Engineering, Chalmers University of Technology, 41296 Gothenburg, Sweden

Overview of Supplementary Material

Table S1: Loss of viability and activity of BB-12 and BB-46, harvested in the exponential or stationary growth phase, during short-term storage.

Data S1: Raw data from differential gene expression analysis: comparison of gene expression of BB-12 between growth phases.

Data S2: Raw data from differential gene expression analysis: comparison of gene expression of BB-46 between growth phases.

Data S3: Raw data from differential gene expression analysis: comparison of regulation of orthologous genes in BB-12 and BB-46 between growth phases.

Table S2: Genes associated with carbohydrate utilization that have been upregulated in BB-12 and/or BB-46 in the stationary phase.

Figure S1: Sequence alignment of L-homoserine O-acetyltransferase gene BCE_5534 of *B. cereus* ATCC 10987 [WP_001121522.1] and putative L-homoserine O-acetyltransferase gene BIF_01336 of *B. animalis* ssp. *lactis* BB-12.

Data S4: Raw data from differential gene expression analysis: comparison of expression of orthologous genes of BB-12 and BB-46 in the exponential phase.

Data S5: Raw data from differential gene expression analysis: comparison of expression of orthologous genes of BB-12 and BB-46 in the stationary phase.

Table S3: Membrane fatty acid profile of BB-12 and BB-46 in exponential (EXP) and stationary (STAT) phase.

Figure S2: Epifluorescence microscopy images of DAPI-stained BB-12 (A) and BB-46 (B).

Table S1: Loss of viability and activity of BB-12 and BB-46, harvested in the exponential or stationary growth phase, during short-term storage. Cells were stored for 7 – 28 days at 8 – 10°C and different storage pH (pH 6.5 – 4.5). Viability losses were determined by comparing CFU counts before and after storage. Losses of activity (membrane integrity) were determined applying flow cytometry with 3,3'-diethyloxacarbocyanine iodide (DiOC) as membrane potential-sensitive dye. The quantification level was. Each data point represents biological triplicates ± standard deviation.

Strain	Harvest time point	Storage conditions	Log_{10} loss of viability [CFU mL^{-1}]	Log_{10} loss of active cells [Cells with membrane potential mL^{-1}]
BB-12	Exponential growth phase	pH 6.5, 7 days	-0.01 ± 0.03	-0.20 ± 0.01
BB-46			1.04 ± 0.41	0.53 (two samples below quantification level)
BB-12	Stationary growth phase	pH 6.5, 7 days	0.07 ± 0.04	-0.03 ± 0.02
BB-46			0.02 ± 0.04	-0.14 ± 0.04
BB-12	Stationary growth phase	pH 5.5, 7 days	0.29 ± 0.07	0.19 ± 0.04
BB-46			0.21 ± 0.05	-0.07 ± 0.06
BB-12	Stationary growth phase	pH 4.5, 7 days	0.42 ± 0.04	0.45 ± 0.02
BB-46			2.01 ± 0.24	0.41 ± 0.27 (one sample below quantification level)
BB-12	Stationary growth phase	pH 6.5, 28 days	0.69 ± 0.19	0.87 ± 0.10
BB-46			9.15 (no survival)	0.88 (two sample below quantification level)

Table S2: Genes associated with carbohydrate utilization that have been upregulated in BB-12 and/or BB-46 in the stationary phase. (ex): extracellular metabolite.

**Gene: encoded enzyme**	**Log_2(FC)**	**Catabolized reaction**
**BB-12**		
BIF_01592 – BIF_01594, BIF_01681: raffinose/melibiose ABC transporter	3.4 – 7.2	ATP + H_2O + melibiose/ raffinose (ex) → ADP + H^+ + P_i + melibiose/ raffinose
BIF_01619, BIF_01620, BIF_00212, BIF_00258, BIF_01681: maltose/short maltodextrins ABC transporter	3.4 – 4.3	ATP + H_2O + maltose/ short maltodextrin (ex) → ADP + H^+ + P_i + maltose/ short maltodextrin
BIF_00311: galactose: or lactose:proton symporter	2.6	galactose/lactose (ex) + H^+(ex) → galactose/lactose + H^+
BIF_01646 (*araA*): L-arabinose isomerase	4.3	L-arabinose → L-ribulose
BIF_01647: L-ribulose-5-phosphate-4-epimerase	4.9	L-ribulose-5-phosphate ↔ D-xylulose-5-phosphate
BIF_01648: L-ribulokinase	3.6	L-ribulose + ATP → L-ribulose 5-phosphate + ADP + H^+
BIF_00829: xylulokinase	2.6	D-xylulose + ATP → D-xylulose-5-phosphate + ADP + H^+
BIF_00857: ribokinase	4.5	D-ribose + ATP → D-ribose-5-phosphate + ADP + H^+
BIF_00525: α-galactosidase	2.4	Hydrolysis of terminal, non-reducing α-D-galactose residues in alpha-D-galactosides, including melibiose and raffinose
BIF_01595: α-galactosidase	3.9	Hydrolysis of terminal, non-reducing α-D-galactose residues in alpha-D-galactosides, including melibiose and raffinose
BIF_00799: β-galactosidase	5.0	Hydrolysis of terminal, non-reducing β-D-galactose residues in beta-D-galactosides, including lactose
BIF_00647: β -galactosidase	2.4	Hydrolysis of terminal, non-reducing β-D-galactose residues in beta-D-galactosides, including lactose
BIF_00869 (*malQ*): 4-α-glucanotransferase	3.1	Transglycosylation of maltose and maltodextrins
BIF_01321: α-D-glucose phosphate-specific phosphoglucomutase	2.2	Glucose-1-phosphate ↔ Glucose-6-phosphate<br>Ribose-1-phosphate ↔ Ribose-5-phosphate
BIF_01597: α-glucosidase	3.9	Hydrolysis of terminal, non-reducing 1,4-linked α-D-glucose residues, e.g., in a maltodextrin, with release of a D-glucose
**BB-46**		
I3242_01825: arabinose:proton symport	2.0	arabinose (ex) + H^+(ex) → arabinose + H^+
I3242_07745: galactose:proton symporter	2.6	galactose (ex) + H^+(ex) galactose + H^+
I3242_01150, I3242_01155: glucose PEP:PTS system	3.2, 2.7	phosphoenolpyruvate + D-glucose → glucose-6-phosphate + pyruvate
I3242_00540: α-glucosidase	2.5	Hydrolysis of terminal, non-reducing 1,4-linked α-D-glucose residues, e.g., in a maltodextrin, with release of a D-glucose
I3242_07765: ribokinase	2.8	D-ribose + ATP → D-ribose-5-phosphate + ADP + H+
I3242_06810: xylulokinase	2.2	D-xylulose + ATP → D-xylulose-5-phosphate + ADP + H+

```
                                     20                     40                     60
                                      |                      |                      |
BCE_5534   MPIIIDKDLP  ARKVLQEENI  FVMTKERAET  QDIRALKIAI  LNLMPTKQET  EAQLLRLIGN  60
BIF_01336  MPIKIPGGLP  ARAILDSERI  FALEKPEAER  QRVRPLKMVI  LNLMPKKIET  ETQLLRLISK  60
Consensus  MPIXIXXXLP  ARXXLXXEXI  FXXXKXXAEX  QXXRXLKXXI  LNLMPXKXET  EXQLLRLIXX

                                     80                    100                    120
                                      |                      |                      |
BCE_5534   TPLQLDVHLL  HMESHLSRNV  AQEHLTSFYK  TFRDIENEKF  DGLIITGAPV  ETLSFEEVDY  120
BIF_01336  SPLQVEIDFM  KTSTHESKHV  SADHLVKFYE  PLEAYEGNCY  DGLIVTGAPV  EHLRFEDVDY  120
Consensus  XPLQXXXXXX  XXXXHXSXXV  XXXHLXXFYX  XXXXXEXXXX  DGLIXTGAPV  EXLXFEXVDY

                                    140                    160                    180
                                      |                      |                      |
BCE_5534   WEELKRIMEY  SKTNVTSTLH  ICWGAQAGLY  HHYGVQKYPL  KEKMFGVFEH  EVREQHVKLL  180
BIF_01336  WDEFKRVLDW  SSTHVFSTMY  MCWGAMAALY  QRYGVHKMDL  DEKLFGVFPQ  FLQDEYCFLT  180
Consensus  WXEXKRXXXX  SXTXVXSTXX  XCWGAXAXLY  XXYGVXKXXL  XEKXFGVFXX  XXXXXXXXLX

                                    200                    220                    240
                                      |                      |                      |
BCE_5534   QGFDELFFAP  HSRHTEVRES  DIREVKELTL  LANSEEAGVH  LVIGQEGRQV  FALGHSEYSC  240
BIF_01336  NGFDEIALQP  HSRLAGVNEA  DIAANPELQV  LTWGPQAGPG  LIATRDFSEV  FALGHWEYGK  240
Consensus  XGFDEXXXXP  HSRXXXVXEX  DIXXXXELXX  LXXXXXAGXX  LXXXXXXXXV  FALGHXEYXX

                                    260                    280                    300
                                      |                      |                      |
BCE_5534   DTLKQEYERD  RDKGL-NIDV  PKNYFKHDNP  NEKPLVRWRS  HGNLLFSNWL  NYYVYQETPY  299
BIF_01336  YTLAEEYRRD  MAKGMSNVPF  PRNYFPNDDP  ALAPLFSWRA  HANLLWRNWL  NW-VYQTTPY  299
Consensus  XTLXXEYXRD  XXKGXSNXXX  PXNYFXXDXP  XXXPLXXWRX  HXNLLXXNWL  NXYVYQXTPY

                                    320                    340
                                      |                      |
BCE_5534   VL--------  ----------  ----------  ----------  ---  301
BIF_01336  DLSEVPQLRA  ERRLGTDRSI  RHAPCSPRDD  QFLPFDTSGY  GLK  342
Consensus  XLSEVPQLRA  ERRLGTDRSI  RHAPCSPRDD  QFLPFDTSGY  GLK
```

Figure S1: Sequence alignment of L-homoserine O-acetyltransferase gene BCE_5534 of *B. cereus* ATCC 10987 [WP_001121522.1] and putative L-homoserine O-acetyltransferase gene BIF_01336 of *B. animalis* ssp. *lactis* BB-12. BIF_01336 was annotated as L-homoserine O-succinyltransferase according to PGAP. However, the gene possess a glutamate at position 111. According to previous studies in *Bacillus cereus* this suggests that the gene actually encodes a homoserine O-acetyltransferase.

Table S3: Membrane fatty acid profile of BB-12 and BB-46 in exponential (EXP) and stationary (STAT) phase. Each data point represents the mean of biological triplicates ± standard deviation. UFA/SFA: unsaturated:saturated fatty acid ratio. The significance of the differences between means was assessed in t-tests. Means with the same superscript letters show differences at $p \leq 0.05$ before multiple testing, while means with the same number of superscript asterisks (*) within the same group show differences at $p_{adj} \leq 0.05$ after correcting for multiple testing. When superscripts are omitted, no significant difference was observed. White background: Fatty acids that are present in small proportions (≤ 5%) and whose proportions varied less than 2% when comparing the fatty acid profile of one strain between growth phases or the profiles of BB-12 and BB-46 at a given growth phase.

**Strain**	**BB-12**		**BB-46**	
**Growth phase**	**EXP**	**STAT**	**EXP**	**STAT**
**Nonanoic acid (C9:0)**	4.5 ± 2.5	1.9 ± 0.8	4.6 ± 2.6	13.1 ± 4.9
**Capric acid (C10:0)**	3.9 ± 0.7^{a}	3.1 ± 1.8	6.4 ± 0.4^{a}	4.7 ± 2.1
**Undecanoic acid (C11:0)**	2.2 ± 1.1	0.8 ± 0.4	2.2 ± 1.1	5.8 ± 2.3
**Undecenoic acid (C11:1)**	0.3 ± 0.2	0.3 ± 0.1	1.1 ± 0.7	0.3 ± 0.1
**Lauric acid (C12:0)**	0.7 ± 0.1	0.7 ± 0.2	0.5 ± 0.0	0.4 ± 0.1
**Dodecenoic acid (C12:1)**	1.9 ± 0.8	0.8 ± 0.4^{a}	2.0 ± 0.9	4.4 ± 1.4^{a}
**Tridecanoic acid (C13:0)**	0.4 ± 0.1	0.2 ± 0.2	0.5 ± 0.1	0.5 ± 0.2
**Tridecenoic acid (C13:1)**	0.8 ± 0.2	0.6 ± 0.1	0.8 ± 0.2	0.6 ± 0.2
**Myristic acid (C14:0)**	2.9 ± 0.3^{a}	4.0 ± 0.1^{a}	1.8 ± 0.7	3.4 ± 1.1
**Myristoleic acid (C14:1)**	0.6 ± 0.1^{a}	0.4 ± 0.2	0.9 ± 0.1^{a}	0.7 ± 0.1
**Pentadecanoic acid (C15:0)**	0.5 ± 0.1^{a}	0.4 ± 0.2^{b}	0.7 ± 0.3^{a}	0.6 ± 0.1^{b}
**Pentadecenoic acid (C15:1)**	0.7 ± 0.1^{b}	0.6 ± 0.2	1.6 ± 0.2^{a,b}	0.9 ± 0.1^{a}
**Palmitic acid (C16:0)**	34.7 ± 2.2^{*,a}	39.9 ± 3.9^{**,a}	18.4 ± 1.6^{*,b}	9.5 ± 2.4^{**,b}
**Palmitelaidic acid (C16:1, T)**	0.9 ± 0.4	1.4 ± 0.1^{a}	1.2 ± 0.3	1.1 ± 0.1^{a}
**Palmitoleic acid (C16:1)**	2.3 ± 0.2	1.4 ± 0.1	1.2 ± 0.3	1.1 ± 0.1
**10-Heptadecenoic acid (C17:1)**	1.1 ± 0.2	1.0 ± 0.4	1.2 ± 0.2	1.1 ± 0.2
**Stearic acid (C18:0)**	7.3 ± 0.3^{a}	9.0 ± 0.5^{a,c}	6.9 ± 0.3^{b}	3.6 ± 1.3^{b,c}
**Oleic acid (C18:1)**	25.4 ± 3.5	23.9 ± 1.8	36.6 ± 5.8	37.6 ± 10.9
**Linoelaidic acid (C18:2TT)**	2.3 ± 0.2^{a}	3.5 ± 0.3^{a,b}	2.0 ± 0.0	2.1 ± 0.3^{b}
**Linoleic acid (C18:2)**	1.3 ± 0.2^{a}	0.9 ± 0.4	2.0 ± 0.2^{a}	1.7 ± 0.5
**γ-linolenic acid (C18:3, n-6)**	1.3 ± 0.1^{*,a}	2.5 ± 0.3^{a,c}	0.7 ± 0.0^{*,b}	0.6 ± 0.1^{b,c}
**α-linolenic acid (C18:3, n-3)**	1.7 ± 0.3^{a}	1.3 ± 0.6	2.5 ±0.2^{a}	2.2 ± 0.6
**Arachidic acid (C20:0)**	2.3 ± 0.6^{a}	1.5 ± 0.7^{a,b}	3.1 ± 0.8	3.3 ± 0.5^{b}
**UFA/SFA**	0.7 ± 0.1^{a}	0.6 ± 0.0	1.2 ± 0.2^{a}	1.2 ± 0.4
**Chain length**	16.0 ± 0.3^{a}	16.3 ± 0.2^{a}	16.1 ± 0.4	15.3 ± 0.8

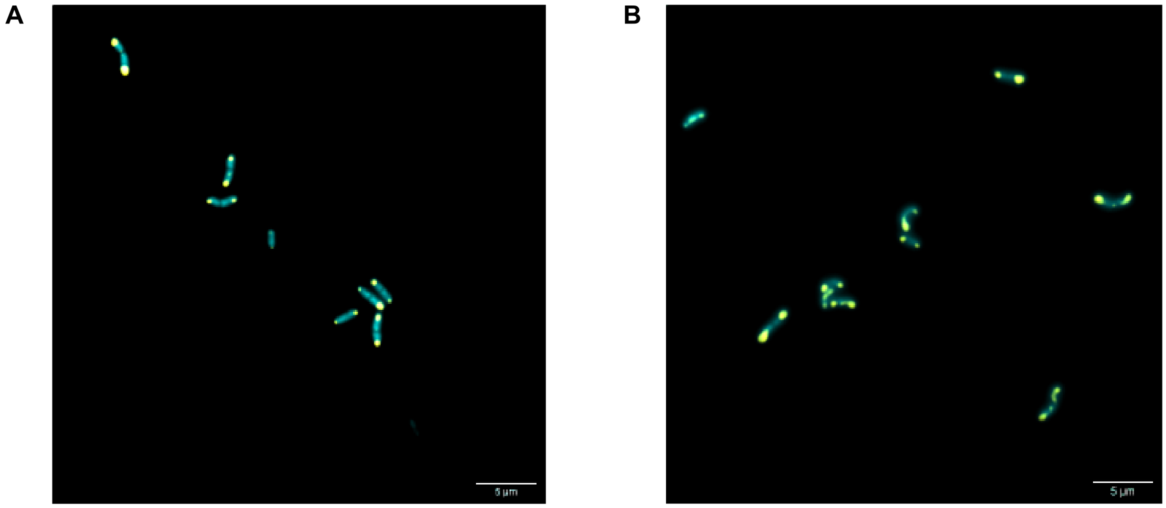

Figure S2: Epifluorescence microscopy images of DAPI-stained BB-12 (A) and BB-46 (B). Cells were fixed with 4% formaldehyde and permeabilized with 0.3% Triton-X 100, before being incubated with DAPI. The images were taken with an epifluorescence microscope. Nucleic acid was visualized at excitation/emission wavelengths of 365 nm/460 nm (blue) and polyphosphate granules were visualized at excitation/emission wavelengths of 469 nm/525 nm (yellow).
